# Identification of broadly neutralizing antibody epitopes in the HIV-1 envelope glycoprotein using evolutionary models

**DOI:** 10.1186/1743-422X-10-347

**Published:** 2013-12-02

**Authors:** Miguel Lacerda, Penny L Moore, Nobubelo K Ngandu, Michael Seaman, Elin S Gray, Ben Murrell, Mohan Krishnamoorthy, Molati Nonyane, Maphuti Madiga, Constantinos Kurt Wibmer, Daniel Sheward, Robert T Bailer, Hongmei Gao, Kelli M Greene, Salim S Abdool Karim, John R Mascola, Bette TM Korber, David C Montefiori, Lynn Morris, Carolyn Williamson, Cathal Seoighe

**Affiliations:** 1 School of Mathematics, Statistics and Applied Mathematics, National University of Ireland Galway, Galway, Ireland; 2 Department of Statistical Sciences, Faculty of Science, University of Cape Town, Cape Town, South Africa; 3 Centre for HIV and STIs, National Institute for Communicable Diseases of the National Health Laboratory Services, Johannesburg, South Africa; 4 School of Pathology, University of the Witwatersrand, Johannesburg, South Africa; 5 Institute of Infectious Diseases and Molecular Medicine, Division of Medical Virology, Faculty of Health Sciences, University of Cape Town and NHLS, Cape Town, South Africa; 6 Beth Israel Deaconess Medical Center, Division of Viral Pathogenesis, Harvard, USA; 7 Biomedical Informatics Research Division, eHealth Research and Innovation Platform, Medical Research Council, Tygerberg, South Africa; 8 Computer Science Division, Department of Mathematical Sciences, University of Stellenbosch, Stellenbosch, South Africa; 9 Los Alamos National Laboratory, Los Alamos, NM, USA; 10 Vaccine Research Center, National Institute of Allergy and Infectious Diseases, NIH, Bethesda, MD, USA; 11 Department of Surgery, Duke University Medical Center, Durham, NC, USA; 12 Centre for the AIDS Programme of Research in South Africa, University of KwaZulu-Natal, Durban, South Africa; 13 Centre for the AIDS Programme of Research in South Africa, Columbia University, Columbia, USA; 14 Santa Fe Institute, Santa Fe, NM, USA

**Keywords:** HIV, Antibodies, Neutralization sensitivity, Epitope prediction, Evolutionary model

## Abstract

**Background:**

Identification of the epitopes targeted by antibodies that can neutralize diverse HIV-1 strains can provide important clues for the design of a preventative vaccine.

**Methods:**

We have developed a computational approach that can identify key amino acids within the HIV-1 envelope glycoprotein that influence sensitivity to broadly cross-neutralizing antibodies. Given a sequence alignment and neutralization titers for a panel of viruses, the method works by fitting a phylogenetic model that allows the amino acid frequencies at each site to depend on neutralization sensitivities. Sites at which viral evolution influences neutralization sensitivity were identified using Bayes factors (BFs) to compare the fit of this model to that of a null model in which sequences evolved independently of antibody sensitivity. Conformational epitopes were identified with a Metropolis algorithm that searched for a cluster of sites with large Bayes factors on the tertiary structure of the viral envelope.

**Results:**

We applied our method to ID_50_ neutralization data generated from seven HIV-1 subtype C serum samples with neutralization breadth that had been tested against a multi-clade panel of 225 pseudoviruses for which envelope sequences were also available. For each sample, between two and four sites were identified that were strongly associated with neutralization sensitivity (2ln(BF) > 6), a subset of which were experimentally confirmed using site-directed mutagenesis.

**Conclusions:**

Our results provide strong support for the use of evolutionary models applied to cross-sectional viral neutralization data to identify the epitopes of serum antibodies that confer neutralization breadth.

## Background

A successful HIV-1 vaccine is likely to require the induction of neutralizing antibodies that can prevent infection. HIV-1 entry into host cells is mediated by the HIV-1 envelope glycoprotein, which forms a trimeric structure on the surface of the virus. Each of these envelope “spikes” consists of three identical, non-covalently associated heterodimers of surface gp120 and transmembrane gp41. Antibodies that bind the envelope can be detected within eight days of infection [1]. However, neutralizing antibodies that specifically bind the trimeric form of the envelope and prevent cell entry are slower to develop and appear about 3–6 months after infection [2-7]. Importantly, these early neutralizing antibodies preferentially bind the autologous virus and are therefore strain-specific [4,5,7-9]. In contrast, recent studies have revealed that 15-20% of infected people are able to develop serum antibodies that exhibit neutralization of genetically diverse HIV-1 strains [10-12]. However, these broadly neutralizing antibodies are generally only produced 2–4 years after infection [12,13]. Although these neutralizing antibodies may not protect against disease progression [12,14], the fact that the host B cell response has the potential to generate such broadly reactive neutralizing antibodies against HIV-1 has led to renewed interest in the development of a preventative vaccine that elicits similar types of antibodies [3].

The HIV-1 envelope has evolved an array of mechanisms that hinder binding by neutralizing antibodies. The envelope glycoprotein is genetically variable, conformationally flexible and heavily glycosylated, resulting in either escape from antibody recognition or shielding of neutralization sensitive sites [4,15,16]. The narrowness of the initial response, together with the plasticity of the envelope protein, allows escape variants to evolve rapidly in the infected individual [4,5,8,17]. Nevertheless, fitness constraints preclude complete resistance and certain regions of envelope remain vulnerable to antibody neutralization [18]. Recently, new highly potent monoclonal antibodies have been isolated that define targets on the HIV envelope. This includes the PG9/PG16 monoclonal antibodies that recognize an epitope involving V2 and V3 created by the trimeric structure [19], the PGT antibodies that mostly rely on a glycan at position 332 in the C3 region of gp120 [20], the VRC01 monoclonal antibody that targets the CD4 binding site [21] and the gp41 membrane proximal external region-specific antibody 10E8 [22]. These broadly neutralizing monoclonal antibodies greatly expand our understanding of the conserved epitopes on the envelope, which were previously defined by IgG1b12 against the CD4 binding site, 2G12 against the glycan shield in the outer domain and 4E10 and 2 F5 that recognize distinct epitopes in the membrane proximal external region of gp41 [23-27].

Broad serum neutralization could potentially be mediated by a polyclonal set of neutralizing antibodies that accumulate over time and target several distinct regions of envelope [13,28]. Alternatively, the gradual focusing of the B cell response onto functionally conserved regions of envelope could be responsible for the potent neutralization of diverse HIV-1 isolates in some individuals. Although it is likely that both scenarios occur in infected subjects [29], the latter possibility is supported by the recent identification of monoclonal antibodies whose neutralization breadth matches that of the corresponding serum [19-21]. The identification and characterization of epitopes that are targeted by antibodies in broadly neutralizing sera is a key step in the design of immunogens that can potentially induce broad neutralizing antibodies against HIV-1.

B cell epitope prediction is complicated by the conformation-dependent nature of antigen-antibody binding. Although more than 90% of antibody epitopes are estimated to be conformational in nature, most experimental and computational methods are designed to identify only linear epitopes [30-32]. Here, we present a novel computational method that uses cross-sectional neutralization sensitivity and sequence data from a large panel of viruses to predict sites that lie within antibody epitopes. Similar data were analyzed by Gnanakaran et al. [15], who developed phylogenetically-corrected methods for identifying signature amino acid sites in envelope that were associated with neutralization phenotypes. While their method required discrete neutralization phenotypes, our method permits the use of continuous neutralization measures, which should enhance statistical power. More recently, Georgiev et al. [33] developed a technique to deconvolute the antibody specificities in polyclonal sera. However, their method does not identify amino acid positions associated with neutralization sensitivity and does not account for the phylogenetic relationships between viruses.

Our method incorporates neutralization sensitivities directly into a phylogenetic model of molecular evolution. Amino acid sites at which the pattern of evolution correlated with changes in neutralization sensitivity across the phylogenetic tree were identified. We hypothesized that many of the sites that were associated with changes in the neutralization sensitivity of multiple viruses lie within antibody epitopes. In order to identify sites in the alignment that were most likely to influence neutralization sensitivity, we used Bayes factors to compare the fit of a model that allows the amino acid frequency at each site to depend on neutralization sensitivities (epitope model) to that of a model which assumes independence (non-epitope model). A contiguous set of sites on the primary sequence that favored the epitope model provided evidence for a B cell epitope (which could be linear or conformational), while a set of sites with large Bayes factors that were clustered in three-dimensional space provided evidence of a conformational epitope. We used this approach to predict epitopes targeted by the broadly neutralizing antibodies present in the sera of seven HIV-1 subtype C-infected individuals enrolled in the CAPRISA Acute Infection study [12]. All sites with strong support were then tested experimentally using mutagenesis studies. We found that our model accurately predicted amino acid residues that were targeted by broadly neutralizing serum antibodies, including those present in V2, the glycan shield and the membrane proximal external region.

## Results

**Figure 1:**
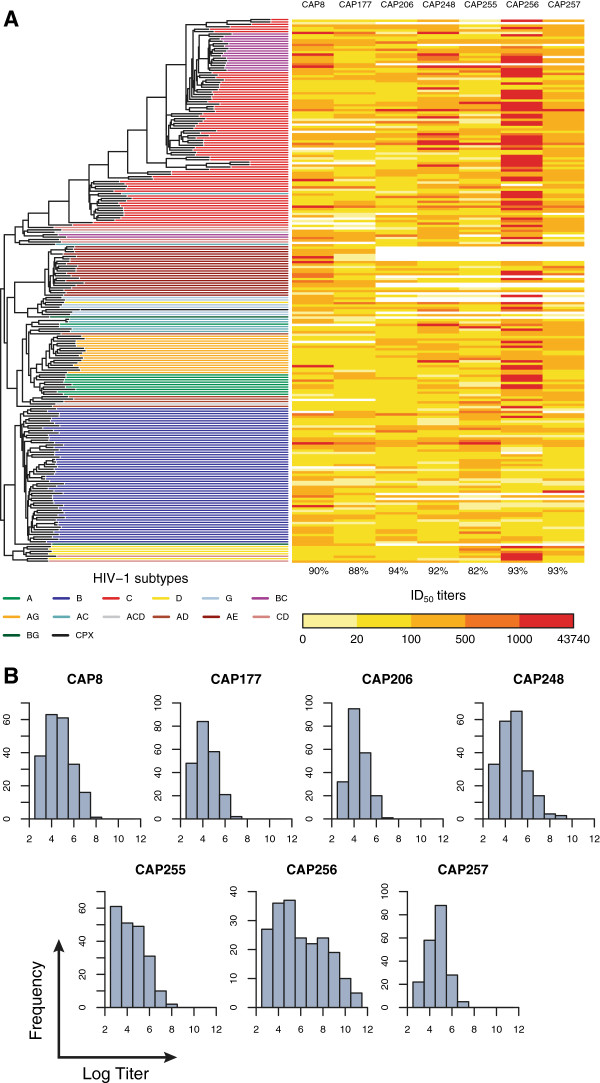
**ID**_**50 **_**titers for each serum sample. (A)** Heat map of ID_50_ titers clustered by the viral phylogeny. The percentages below the heat map indicate the percentage of panel viruses that were neutralized (ID_50_ > 20) by the antibodies in each serum. **(B)** Histograms of the natural logarithm of ID_50_ titer. The distribution for CAP256 was notably different from that of the other sera.

We have previously identified sera from seven HIV-1 subtype C-infected women in the CAPRISA 002 cohort that showed substantial neutralization breadth at 3 years post-infection against a panel of 42 viruses from subtypes A, B and C [12]. In order to assess neutralization serotypes more comprehensively (as part of the Neutralization Serotype Discovery Project of the Collaboration for AIDS Vaccine Discovery), these sera were tested against a much larger panel of 225 envelope-pseudotyped viruses, including subtypes A (*n* = 10), B (*n* = 56), C (*n* = 69), D (*n* = 6) and G (*n* = 6), as well as several circulating recombinant forms (CRFs) (*n* = 78). For each pseudovirus, the neutralization titer of a subject’s serum was recorded as the reciprocal of the maximal plasma dilution that could inhibit 50% of viral entry (denoted as ID_50_). The neutralization breadth, as measured by the overall percentage of viruses neutralized (ID_50_ > 20) by each serum, ranged from 82% (CAP255) to 94% (CAP206) (Figure 1A). Notably, the titers of CAP256 (which neutralized 93% of the panel viruses) were considerably higher than those of any other serum, with an ID_50_ exceeding 10, 000 against some viruses (Figure 1B). Serum from CAP8, CAP177, CAP206 and CAP255 showed no clear subtype specificity. In contrast, the CAP248, CAP256 and CAP257 sera neutralized subtype C viruses better than the subtype B panel viruses (Wilcoxon tests of median titer, *p* < 0.001). Since our method takes the phylogenetic relationships between the panel viruses into account, the preferential neutralization of certain HIV-1 clades by these serum antibodies did not bias our results.

### Identification of amino acid residues targeted by the broadly neutralizing serum of CAP256

We first analyzed the data from CAP256 which showed the highest neutralization titers (Figure 1) mainly attributed to antibodies targeting a PG9/PG16-like quaternary (trimer-specific) epitope [34]. These antibodies were shown to be dependent on the R166 and K169 residues in the V2 region with F159, N160, L165, D167, and K171 also contributing to the epitope, though to a lesser extent. Consequently, these data provided a set of well-characterized true-positive sites that could be used to test whether our method had the capacity to detect key sites within neutralizing antibody epitopes.

Given a coding sequence alignment and a phylogenetic tree for the virus panel, we used evolutionary models to identify sites in the alignment at which the pattern of evolution along branches of the tree was dependent on the neutralization titer of the virus at the tip of each branch (see Methods for details). Scaled Bayes factors (denoted as *B*_*k*_ for HXB2 amino acid position *k*) were used to compare the fit of a model where the equilibrium frequency of the “reference” amino acid at each site depended on neutralization titer to that of a null model of independence. The reference amino acid at each site was defined by identifying a “reference sequence” that was sensitive to the antibodies present in a particular serum sample. Because our model predictions depended on the reference amino acid at each site, several reference sequences were considered for each serum.

**Figure 2:**
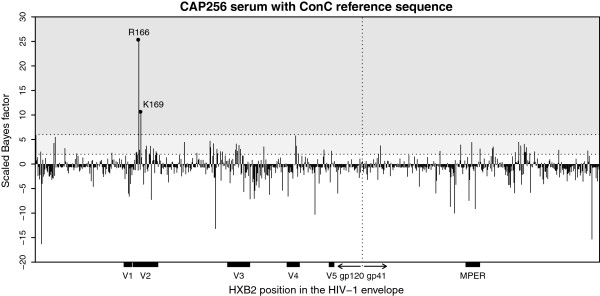
**Scaled Bayes factors for CAP256.** Neutralization titers were strongly associated with sites 166 and 169 when the ConC reference sequence was used. These sites have previously been shown to contribute significantly to the epitope targeted by CAP256 antibodies [12]. Shaded regions indicate the degree of evidence for an association with ID_50_: white indicates no or negligible evidence, light grey indicates moderate evidence and dark grey indicates strong evidence [55]. Sites that were strongly associated with ID_50_ are annotated with their HXB2 position and the amino acid found to be enriched among sensitive (high titer) viruses at that site.

Our model provided striking evidence of an association between neutralization sensitivity and the amino acids present at sites 166 (*B*_166_ = 25.4) and 169 (*B*_169_ = 10.7) (see Figure 2). Both of these sites are critical for the formation of the quaternary epitope targeted by the serum antibodies of CAP256 [34]. In particular, we found that viruses with an arginine at position 166 or a lysine at position 169 were significantly more sensitive to CAP256 serum neutralization than viruses with other amino acid residues at these positions, supporting previous studies [34]. Use of three other reference sequences identified the same 2 residues (Additional file 1: Figures S1A-C). Experimental confirmation for the role of 166R and 169K in CAP256 neutralization is shown in Table 1 where mutations at both sites resulted in major reductions in neutralization titers, particularly in the CAP45 backbone.

**Table 1:** All sites with scaled Bayes factors ≥ 6

**Serum**	**HXB2 position**	**Reference residue**^**‡**^	**Scaled Bayes factor (LFDR)**	**Backbone**	**ID**_**50 **_**fold effect**
CAP256	166^†^	Arg	25.4 (0.0001)	CAP45	261.3*
				ConC	22.2*
	169^†^	Lys	10.7 (0.083)	CAP45	365.8*
				ConC	15.3*
CAP8	24	Met	6.4 (1.000)		NT
	295	Asn	11.9 (1.000)	TRO	0.4
	316^†^	Thr	9.1 (1.000)	ConC	1.8
	535	Ile	6.0 (1.000)		NT
CAP257	166	Arg	6.3 (0.738)	ConC	1.8
	295	Asn	7.0 (0.665)	Q842	0.1
	648	Glu	6.1 (0.757)	Du156	1.2
				RHPA	2.9*
	702	Leu	6.2 (0.747)		NT
CAP255	332	Asn	8.0 (0.622)	Q23	4.0*
				TRO	2.9*
				ConC	0.2
	334	Ser	6.8 (0.750)	TRO	>12.4*
				ConC	0.3
	351	Glu	6.8 (0.750)		NT
	837	Phe	10.1 (0.366)		NT
CAP177	209	Thr	8.8 (0.388)	TRO	0.4
	332^†^	Asn	7.3 (0.573)	ConC	1.7
				Q23	11.0*
				TRO	>2.8*
	334^†^	Ser	7.8 (0.511)	ConC	2.1*
				TRO	0.2
	683	Lys	6.3 (0.689)		NT
CAP206	150	Met	6.7 (0.457)		NT
	655	Lys	7.3 (0.384)		NT
CAP248	85	Val	6.5 (1.000)		NT
	340	Glu	6.0 (1.000)	ConC	2.2*
				CAP45	0.6
	651	Asn	7.4 (1.000)	ConC	2.0*
				CAP45	1.3
				Du156	2.4*
	659	Asp	8.9 (1.000)	ConC	2.9*
				CAP45	0.5

^†^Sites with *q* ≤ 1/3 based on the method of Gnanakaran et al. [15]. ^‡^Amino acid found to be significantly enriched among sensitive (high titer) viruses based on our evolutionary model. *Fold effect ≥ 2. NT = Not tested.

We also found weak evidence of an association with neutralization sensitivity at sites 162, 163, 167, 176, 177, 181, 183, 187, 193 and 194 in V2 (2 < *B*_*k*_ < 6). The fact that our model assigns moderately large scaled Bayes factors to this cluster of sites is encouraging. However, there were many other sites throughout gp120 and gp41 with scaled Bayes factors in this intermediate region (2 < *B*_*k*_ < 6), as might be expected when multiple model comparisons are performed. To account for the fact that multiple model comparisons were carried out, we computed the local false discovery rate (LFDR) associated with the Bayes factor at each site (see Methods). As expected, the large Bayes factors at sites 166 and 169 were highly likely to be true positives with low LFDRs of 0.0001 and 0.083, respectively (Table 1). The next highest scaled Bayes factor was 5.95 (LFDR = 0.492) at site 2 when the reference sequence contained an arginine at this position. Since this scaled Bayes factor was much smaller than those at positions 166 and 169 and below our significance threshold of 6, we did not regard any other amino acid positions as significantly associated with neutralization titer.

### Prediction of sites targeted by PG9/PG16-like antibodies

In order to explore the utility of our model, we tested two additional sera predicted to have a similar specificity to CAP256, but with considerably lower titers. Four sites were found to be strongly associated (*B*_*k*_ ≥ 6) with neutralization by CAP8 serum antibodies (see the scaled Bayes factors in Table 1; plots shown in Additional file 1: Figure S2). The largest scaled Bayes factor of 11.9 was observed at position 295, a potential N-linked glycosylation (PNG) site. However, replacing the asparagine at this position in the TRO backbone did not reduce ID_50_ titers. Site 316 in V3 was also found to be significantly associated with neutralization sensitivity (*B*_316_ = 9.1). Mutation of this residue in the ConC backbone resulted in a large drop in ID_50_ titers from 11,000 to 6,000. Mutations in the V3 region are known to modulate neutralization sensitivity of the conserved V2 epitope recognized by PG9/PG16-like antibodies. Therefore, although no sites within the conserved C-strand in V2 were detected, the detection of residue 316 in V3 was consistent with a known neutralizing specificity in the CAP8 serum, which was previously shown to target a PG9/PG16-like trimer-specific epitope [12,35]. Sites 24 (*B*_24_ = 6.4) in the envelope signal peptide and site 535 (*B*_535_ = 6.0) in gp41 were also found to be associated with titer, though we do not expect these to contribute to CAP8 antibody neutralization and therefore did not test them experimentally.

For CAP257 serum, we identified signals at positions 166 (*B*_166_ = 6.3) and 295 (*B*_295_ = 7.0) in the V2 and V3 regions of gp120, respectively, and at positions 648 (*B*_648_ = 6.1) and 702 (*B*_702_ = 6.2) in gp41. Sites 166, 295 and 648 were tested with site-directed mutagenesis and the substitution of alanine at site 648 in the RHPA backbone was found to reduce ID_50_ titers by more than 2 fold (Table 1). Serum from this individual has previously been shown to contain at least two distinct antibody specificities; one targeting a PG9/PG16-like trimer-specific epitope, followed by a second that overlaps the CD4 binding site (Wibmer et al., submitted). Although our evolutionary model did identify position 166 in the V2 region, this could not be experimentally validated (Table 1 and Additional file 1: Figure S3). However, introduction of an R166A mutation completely abrogated neutralization by serum from an earlier time point, confirming the existence of a PG9/PG16-like specificity in CAP257 (Wibmer et al., submitted).

### Prediction of sites associated with N332-dependent broadly neutralizing antibodies

**Figure 3:**
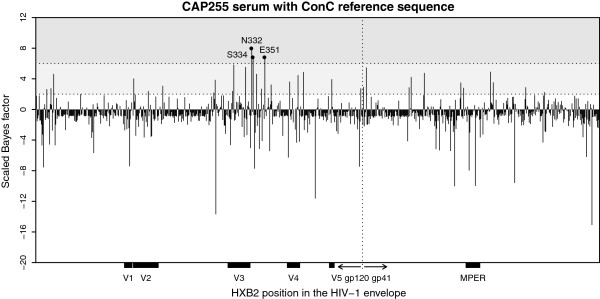
**Scaled Bayes factors for CAP255.** There was strong evidence of an association with ID_50_ titer at sites 332, 334 and 351 when the ConC reference sequence was used. The predictions at sites 332 and 334 were tested and validated experimentally (see Table 1). There were several sites surrounding these three positions in the C3 region that were moderately associated with neutralization sensitivity. The background shading is described in the legend of Figure 2.

Two CAPRISA sera that targeted another broadly neutralizing antibody epitope were tested with our model. The Bayes factors for CAP255 serum identified three sites in the C3 region of gp120 (sites 332, 334 and 351) (see Figure 3; plots for alternative reference sequences are provided in Additional file 1: Figure S4). Two of these sites in close proximity on the primary sequence were strongly associated (*B*_*k*_ ≥ 6) with neutralization titer, namely positions 332 and 334. For site 332, all of the reference sequences contained an asparagine at this position and yielded a large scaled Bayes factor of 8.0, suggesting that viruses with this amino acid were sensitive to CAP255 antibody neutralization. This result was supported by site-directed mutagenesis in the Q23 (4.0 fold) and TRO (2.9 fold) envelope backbones but not in ConC. Our model predictions also showed a large scaled Bayes factor of 6.8 at site 334, which forms part of the same N-linked glycosylation motif, when the reference residue was serine. The involvement of this site was confirmed experimentally in the TRO envelope backbone (>12.4 fold) only. The Q23 reference sequence has a threonine at this position which yields a Bayes factor close to zero, suggesting that this amino acid is not enriched among the sensitive viruses in the panel. This is perhaps surprising, given that a threonine at this position also permits the attachment of an N-linked glycan at site 332. The identification of 332 and 334 as contributing to the CAP255 epitope confirms previous mapping data showing that these antibodies are dependent on the N332 glycan in the C3 region [12], similar to many of the recently described PGT monoclonal antibodies [20].

In addition to sites 332 and 334, we also obtained a large scaled Bayes factor of 6.82 at position 351 when this site contained an isoleucine in the reference sequence for the CAP255 data. Our model also predicted site 837 in gp41. While we do not expect that this position lies within a CAP255 epitope, it is possible that amino acid changes in gp41 contribute to neutralization sensitivity by influencing epitope accessibility through conformational changes to the envelope complex [36,37].

**Figure 4:**
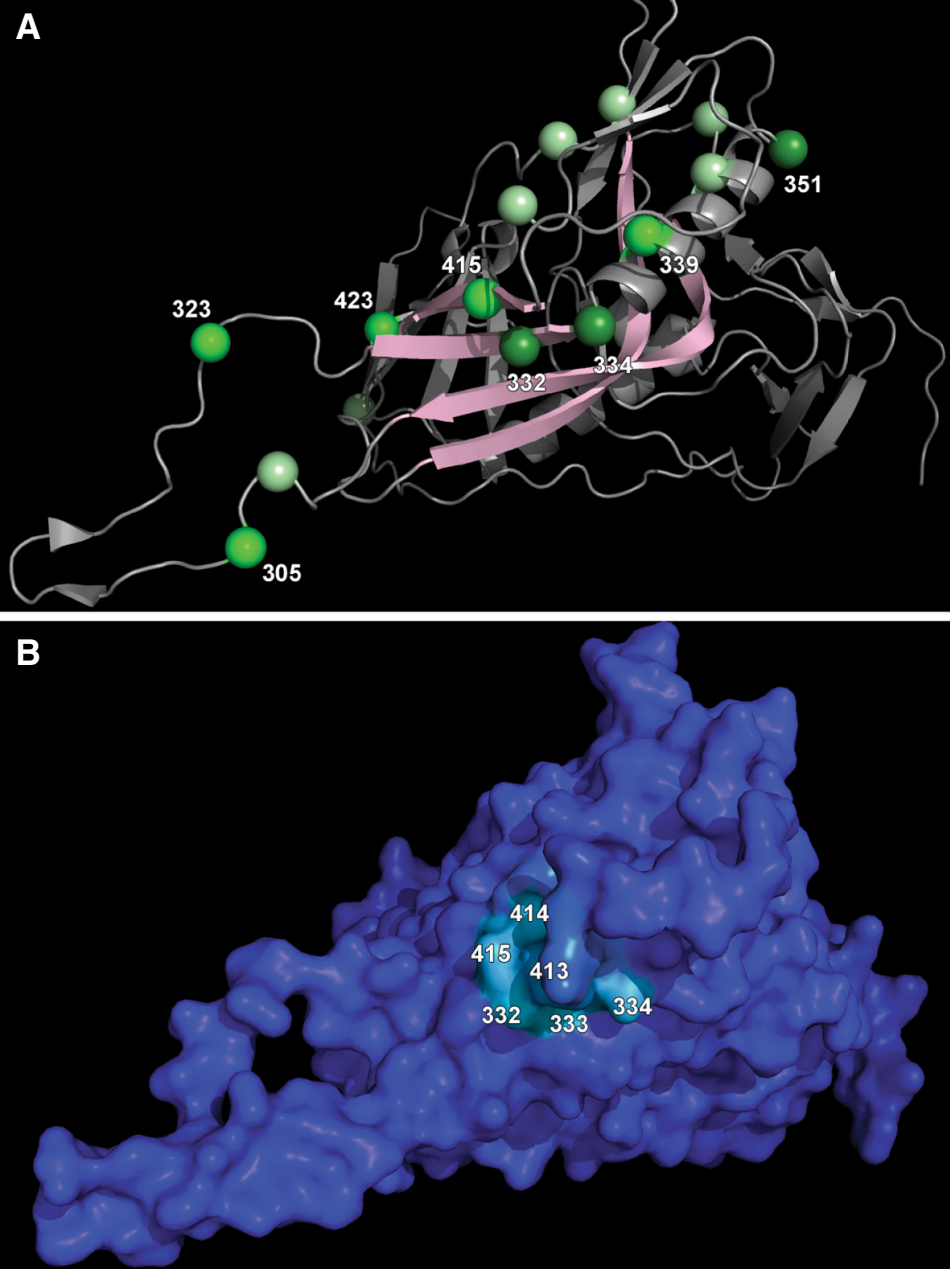
**Three-dimensional predictions for CAP255. (A)** Amino acid residues that were weakly (2 ≤ *B*_*k*_ < 4), moderately (4 ≤ *B*_*k*_ < 6) and strongly (*B*_*k*_ ≥ 6) associated with ID_50_ titer using the ConC reference sequence are shown with light, intermediate and dark green, respectively. There is evidence for a cluster of sites with moderately large Bayes factors on the three-dimensional surface, as might be expected of a B cell epitope. **(B)** Posterior probabilities of a conformational epitope using the three-dimensional Metropolis algorithm. The surface of the protein (PDB ID: 2B4C) was shaded from dark blue (posterior probability = 0) to red (posterior probability = 1) according to the posterior probability assigned to each amino acid residue. There was evidence for a conformational epitope involving the C3 region (residues in the light blue region have posterior probabilities of approximately 0.2).

In addition to the three sites in the C3 region with *B*_*k*_ ≥ 6, several surrounding sites were weakly associated with CAP255 neutralization sensitivity and had 2 < *B*_*k*_ < 6 for at least one reference sequence (see Figure 3). Many of these residues were in close proximity on the tertiary structure as illustrated in Figure 4A. Indeed, residues in this spatial region were found to have higher posterior probabilities of belonging to a conformational epitope after applying our three-dimensional epitope prediction algorithm (see Figure 4B). Site 332 had the highest posterior probability of 0.191 and neighboring residues had slightly lower posterior probabilities.

**Figure 5:**
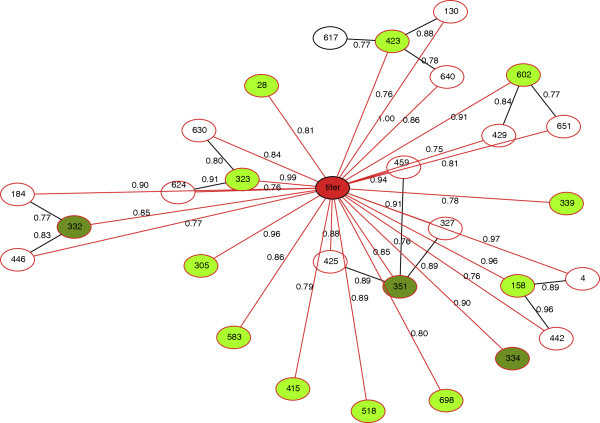
**Bayesian evolutionary-network model for CAP255 [**[38]**].** The red node corresponds to ID_50_ titer and all other nodes represent sites in the HIV-1 envelope. Nodes with scaled Bayes factors > 6 are shaded in dark green, while nodes with scaled Bayes factors between 2 and 6 are shaded in light green. An edge connecting two nodes indicates that there is a direct association between the two nodes. Edges are labeled with the estimated posterior probability of an interaction between the nodes they connect. Only sites with scaled Bayes factors > 4 or posterior probabilities of an association with such a site > 0.75 are shown. Since all of the sites with Bayes factors > 4 are directly connected to the ID_50_ node, none of the predicted associations could be attributed to compensatory mutations.

It is possible that the association with neutralization sensitivity at some of these spatially-proximal sites might be a consequence of compensatory mutations. To investigate this, we used the Bayesian graphical model of Poon et al. [38] to tease apart direct and indirect correlations between residue positions and ID_50_ titer. A network graph indicating the direct associations (with posterior probabilities > 0.75) of all sites with *B*_*k*_ > 4 using the CAP255 data is shown in Figure 5. We found that all of these sites were directly associated with ID_50_ titer and therefore that none of our predicted associations were likely to be due to compensation for resistance-imparting mutations elsewhere. Resistance to CAP255 serum could therefore be attributed to mutations at several sites that either constitute the binding interface of a single antibody or represent independent targets of multiple antibodies. This was the case for all sites with *B*_*k*_ > 6 across all sera (see the supporting information for each serum).

For CAP177 serum, our model predicted four sites that influenced neutralization sensitivity with strong support (*B*_*k*_ ≥ 6). These included sites 209 in the C2 region, 332 and 334 in the C3 region and 683 in the membrane proximal external region of gp41 (Table 1, Additional file 1: Figure S5). Asparagine was observed at a significantly higher frequency at position 332 among neutralization-sensitive viruses than among neutralization-resistant viruses, similar to CAP255 (*B*_332_ = 7.3). This result was experimentally validated in the Q23, TRO and, to a lesser extent, ConC envelope backbones (see Table 1). Our model also predicted that a serine residue at site 334, which forms part of the same N-linked glycosylation motif, imparts neutralization sensitivity, with a large scaled Bayes factor of 7.8 observed at this position. Replacing the serine residue with an alanine in the ConC backbone resulted in a >2 fold decrease in ID_50_ titer, although this same mutation produced a five-fold increase in ID_50_ titers with the TRO backbone. Nonetheless, these experimental results confirmed the importance of this site as a determinant of neutralization titer. This is in line with experimental mapping studies in CAP177, which, like CAP255, showed the presence of antibodies recognizing 332-dependent PGT-like epitopes, though these differ subtly from those in CAP255 in their dependence on variable loops [12,35]. There was, however, less evidence of an association with titer at spatially-proximal sites and consequently our three-dimensional epitope prediction method did not identify a similar region of high posterior probabilities for CAP177 as for CAP255. The prediction that position 209 (*B*_209_ = 8.8) influenced neutralization sensitivity to CAP177 sera could not be experimentally validated in the envelope backbone tested here. We did not attempt to validate site 683 (*B*_683_ = 6.3).

Overall, our model was very effective in predicting sites associated with sensitivity to PGT-like antibodies that are dependent on the glycan at position 332 in both CAP255 and CAP177. These specificities appear to be relatively common among broadly neutralizing sera [19,29,33,39], although strain-specific N332-dependent antibodies have also been reported [40].

### Identification of sites forming part of epitopes in gp41

For CAP206, our model predicted two amino acid positions that were strongly associated with antibody neutralization across six different reference sequences (see Table 1 and Additional file 1: Figure S6). One of these predictions, site 150 (*B*_150_ = 6.7), was in the V1 region, while the other prediction, site 655 (*B*_655_ = 7.3), was near the membrane proximal external region in gp41. The latter prediction is consistent with available mapping data, showing that neutralization breadth in CAP206 is mediated largely by antibodies targeting this region [39]. This is further supported by the recent isolation from CAP206 memory B cells of the moderately broad monoclonal antibody, CAP206-CH12, which recognizes an epitope in the membrane proximal external region [41].

For the final serum examined, CAP248, mapping studies have shown that the serum antibodies target a quaternary epitope that has not yet been defined, but is not PG9/PG16-like [12]. We found associations between the amino acids at positions 651 (*B*_651_ = 7.4) and 659 (*B*_659_ = 8.9) adjacent to the membrane proximal external region of gp41 and neutralization sensitivity (Table 1 and Additional file 1: Figure S7). In particular, our model revealed that viruses with an asparagine at position 651 or an aspartic acid at position 659 exhibited greater sensitivity to CAP248 antibody neutralization than other viruses. Interestingly, both sites lie between two gp41 positions that are known to affect antibody binding to epitopes in gp120 [36,37]. Both of these predictions were confirmed experimentally (see Table 1). Our evolutionary model also identified sites 85 (*B*_85_ = 6.5) and 340 (*B*_340_ = 6.0) in gp120 as significantly associated with titer. The prediction at position 340 was confirmed by mutagenesis, while site 85 was not tested experimentally.

## Discussion

We have developed a novel computational approach to identify amino acid residues in HIV-1 envelope glycoproteins that are targeted by serum neutralizing antibodies. The method can be used to identify neutralizing antibody epitopes when neutralization sensitivity to the serum is available for a large panel of sequenced viruses. Such data will become increasingly available as large-scale efforts to investigate HIV-1 neutralization serotypes are undertaken. The method is an extension of the evolutionary model of Lacerda et al. [42] and allows the distribution of amino acids at each site to depend on neutralization sensitivities at the tips of the branches along a phylogeny. Bayes factors were used to assess the fit of this model at each site relative to that of a model in which the virus evolves independently of the antibody response. Large Bayes factors indicated positions in the alignment where neutralization sensitivity was significantly associated with amino acid composition after accounting for the phylogeny.

This method was applied to neutralization datasets of seven HIV-1 subtype C serum samples that were previously shown to have neutralization breadth [12]. In addition, epitope mapping data was available for six of the seven samples [12,34,39], providing an opportunity to compare our computational method with experimental approaches to epitope mapping. For each serum sample, envelope sequences and neutralization sensitivities at ID_50_ were available from a multi-clade panel of 225 pseudoviruses. Our model identified two to four sites per sample and 24 predictions across all sera that were strongly associated with neutralization sensitivity. We were able to confirm ten of the fifteen sites that we tested using site-directed mutagenesis. In many cases, these corresponded to sites that had previously been linked to antibody neutralization in these and other broadly neutralizing sera. This included two positions in the V2 region (166 and 169) that contributed to a trimer-specific PG9/PG16-like epitope [34] and the glycan at site 332 that is crucial for the epitope targeted by many of the PGT monoclonal antibodies [12,20,35]. We also identified two novel sites near the membrane proximal external region in gp41 that influenced neutralization sensitivity to the CAP248 sera and another two positions in the same region that are associated with sensitivity to CAP206 and CAP257 antibodies. The CAP257 serum was unusual in that neutralization breadth has been shown to be mediated by at least two distinct specificities, which arose sequentially and then waned (Wibmer et al., submitted). The three-year time point, which was used for this study, fell between two specificities when the titers for each were lower than their peak. Nonetheless, our model found a significant association with titer at position 166, which is consistent with the PG9/PG16-like specificity that developed initially.

Our model is similar to the one developed by Gnanakaran et al. [15] who applied the phylogenetically-corrected method of Bhattacharya et al. [43] to identify signature positions in the HIV-1 envelope that were associated with antibody neutralization. While their contingency table-based approach requires that viruses be classified into discrete neutralization phenotypes, our evolutionary model permits the use of continuous neutralization measures, which is likely to enhance the statistical power to detect an association. In addition, our method naturally accounts for the uncertainty of the unknown ancestral sequences, while their approach treats the reconstructed sequences as observed data. In contrast to our current model implementation, the method of Gnanakaran et al. [15] does not require the specification of a neutralization sensitive sequence for each serum sample and can be used to identify combinations of amino acids and sites that influence antibody neutralization sensitivity. Although such combinations could be accommodated straightforwardly in our model, it would be computationally expensive to do so and the increase in the number of tests performed could greatly reduce statistical power. Instead, we identified a set of potentially sensitive amino acid residues at each site based on a selection of pseudoviruses (reference sequences) that have previously been shown to be sensitive to the antibodies of a particular serum sample.

For comparison with our results, we applied the method of Gnanakaran et al. [15] to our neutralization datasets. Of the 24 associations that we identified across the seven sera (see Table 1), five were also detected with the method of Gnanakaran et al. [15] using a *q*-value threshold of 1/3 and three alternative neutralization classifications (see Additional file 2: Table S3). We were able to detect four of our fifteen experimentally-validated associations using this technique. The method of Gnanakaran et al. [15] yielded a further thirteen predictions with *q* ≤ 1/3 across the seven sera, though we did not attempt to validate these experimentally.

The effects of experimental amino acid mutations in different backbones were highly variable, as has been observed in numerous previous studies [12,20,29,34]. This reflects the extremely complex nature of neutralization escape. Our evolutionary model makes the simplifying assumption that each site evolves independently and, as such, does not account for context-specific effects that may alter substitution rates differentially in distinct viruses. This assumption does not hold *in vitro* where the fold effect of mutagenesis depends not only on the mutated residue, but also on the entire backbone context. In addition to mutations directly within epitopes, neutralization resistance may also be meditated by steric shielding of epitopes and distal changes, which may be compensatory or may drive conformational changes that limit accessibility of antibodies to epitopes. While our model may detect non-causal associations between titer and the amino acid composition at sites that experience such compensatory mutations, it is not expected that these associations will be confirmed by mutagenesis. In order to assess the extent to which this was the case, we implemented the evolutionary network model of Poon et al. [38]. Across all seven sera, we found that all sites with scaled Bayes factors greater than six directly coevolved with titer, indicating that our predictions corresponded to potential resistance sites and not compensatory mutations.

To account for multiple testing, we computed the local false discovery rate (LFDR) associated with each of our predictions; that is, the probability that a site is incorrectly found to be associated with titer given the codon data at the site and the prior probability of no association (see Methods). To compute the prior probability of no association, we first estimated the probability of obtaining a positive scaled Bayes factor when no association exists by randomly shuffling the assignment of titers to sequences. For computational reasons, we were only able to perform 100 such permutations. We then made the conservative assumption that a site that is truly associated with titer will always have a positive scaled Bayes factor. Consequently, the LFDR estimates reported in Table 1 should be interpreted as upper bounds on the true LFDRs. As such, many of these estimates are quite large. Indeed, the estimates of the LFDRs for all CAP8 and CAP248 predictions were 1, since the conservative prior probability of no association was 1 for both of these sera. The true LFDRs for these predictions will likely be less than this.

To identify amino acid positions that collectively provide support for the existence of a conformational epitope, we introduced a model in which all sites within a sphere on the tertiary structure evolved according to the epitope model, while the evolution of all other sites was described by the non-epitope model. A Metropolis algorithm was used to explore the posterior density of the center and radius of the sphere and thereby determine the most likely location and size of a conformational epitope on the envelope structure. This approach predicted a known epitope in the C3 region targeted by the CAP255 neutralizing serum. No three-dimensional clusters with high posterior probability were predicted in CAP177, CAP248 or CAP206. The three-dimensional prediction algorithm did, however, find evidence for an epitope targeted by the CAP256, CAP8 and CAP257 antibodies in the V3 region of gp120 (largest posterior probability of 0.367, 0.654 and 0.361, respectively; see Additional file 1: Figures S1E, S2E and S3D). A more realistic model would treat a B cell epitope as a patch of surface-exposed amino acids and estimate the location, shape and size of this surface area. Additional structures including the scaffolded V2 structure [44] and particularly future structures of trimeric forms of the envelope would greatly enhance the power of this approach.

The unobserved titers at all ancestral nodes represent a missing data problem. Unfortunately, integration over all of these unknown variables is not a computationally feasible option for large phylogenies. Instead, one must resort to an imputation procedure. In the model presented here, the ancestral nodes were all assigned the median value of the observed titers at the tips of the phylogeny. We obtained very similar results when codon evolution along the internal branches was modeled with a standard, titer-independent MG94×HKY85 model. Although the internal branches alone cannot provide evidence of an association with neutralization sensitivity in our current model implementation, they are informative about the parameters of the evolutionary model and may therefore increase the power of our method to detect associations based on evolution along the terminal branches. This further distinguishes our approach from other phylogenetically-corrected methods, such as that of Gnanakaran et al. [15], that only consider mutational patterns along the terminal branches. An alternative approach is to reconstruct the ancestral titers based on a model of titer evolution. We investigated this strategy using the ancestral reconstruction method of Felsenstein [45]. Briefly, the ancestral titer at each parent node in the phylogeny was imputed as the average of the observed and estimated titers at its two daughter nodes, weighted by the reciprocal of their branch lengths after accounting for the variability of the titer estimates. We obtained many additional predictions using this procedure, presumably because the inferred ancestral titers provide more information than the median and are treated as if observed without error (see Additional file 2: Table S4 and Additional file 1: Figure S8). We therefore consider our use of median titers at the ancestral nodes as conservative. Existing models of quantitative phenotype evolution, including the Brownian motion-based model of Felsenstein [45] and the Ornstein-Uhlenbeck process [46], assume continuous sample paths and are therefore not appropriate for modeling neutralization titer that undergoes abrupt and discontinuous changes in equilibrium level induced by specific amino acid substitutions. Modeling the evolution of quantitative traits with discontinuous paths is an active area of research [47]. A more realistic model of titer evolution may enhance the statistical power of our method by providing further evidence of genotype/phenotype associations along internal branches of the phylogeny. Our model can readily accommodate ancestral titers inferred by any means and we are currently investigating alternative methods for inferring these unknown titers.

To our knowledge, this is the first attempt to identify conformational B cell epitopes on a tertiary structure based on the evolutionary history of a panel of viruses. In principle, our model could be combined with other structure- and mimotope-based methods that assign scores to residues based on their structural properties and peptide-binding affinities.

## Conclusions

Our method is an effective tool for detecting sequence positions that contribute to neutralizing antibody sensitivity, even within quaternary epitopes on the trimeric envelope complex. However, in the absence of information on the host immune responses experienced by each of the viruses in the panel, we cannot determine whether the correlations between amino acid frequencies and neutralization phenotype at these sites are a consequence of selective immune pressure or random genetic drift. Furthermore, sites that influence neutralization sensitivity through insertions and deletions that alter epitope binding affinity or through shifts in glycosylation patterns will not be detected by our approach [4,8]. Nonetheless, our results provide strong support for the use of evolutionary models as a means to identify key residues in complex B cell epitopes. The conformational nature of these epitopes renders them difficult to discover with currently available computational tools.

## Methods

### Ethics statement

The CAPRISA Acute Infection study was reviewed and approved by the research ethics committees of the University of KwaZulu-Natal (E013/04), the University of Cape Town (025/2004) and the University of the Witwatersrand (MM040202). All participants provided written informed consent for study participation.

### Neutralization and sequence data

We analyzed neutralization datasets from seven HIV-1 subtype C-infected women from the CAPRISA 002 Acute Infection Study [48]. These subjects, namely CAP8, CAP177, CAP206, CAP248, CAP255, CAP256 and CAP257, all developed broadly cross-neutralizing antibodies against HIV-1 [12]. The potency of the serum antibodies collected at 3 years post-infection from each subject was measured against a multiclade panel of 225 envelope-pseudotyped viruses as part of the Neutralization Serotype Discovery Project (NSDP) (see Figure 1A). For each pseudovirus, the neutralization titer of a subject’s serum was recorded as the reciprocal of the maximal plasma dilution that could inhibit 50% of viral entry (ID_50_). The data for CAP256 was used to decide on the optimal modeling strategy.

HIV-1 gp160 sequences from 225 panel viruses were codon aligned with the hidden Markov model implemented in the HIVAlign tool of the Los Alamos National Laboratory (LANL) HIV database (http://www.hiv.lanl.gov/content/sequence/HIV/HIVTools.html). Nucleotide sites at which more than 10% of the sequences contained gaps were removed by deleting the corresponding codon. In doing so, we restricted our analyses to the regions of envelope that are readily aligned [15]. The resulting alignment contained 818 codons, spanning HXB2 envelope positions 1 to 856, excluding sites 13–16, 31, 137–149, 189–190, 310–311, 354–355, 395–408, 460–462 and 514, and including four insertions relative to HXB2. The HIV-1 gp160 alignment is provided as Additional file 3: Dataset S1. The Genbank accession numbers and neutralization titers for the envelope sequences are provided in Additional file 4: Dataset S2.

The alignment was screened for recombination using GARD [49] with a general time reversible nucleotide model and among-site rate variation modeled with a three-category general discrete distribution. A single recombination breakpoint was detected at nucleotide position 1331. Although recombination can be accommodated straightforwardly in our modeling approach by using different phylogenies for each non-recombinant fragment, this would have required the estimation of 447 additional branch length parameters. Rather than attempt this, we conducted a simulation study to assess whether a single recombination breakpoint was likely to substantively affect our results. Briefly, we simulated 10 alignments with a single recombination breakpoint using NetRecodon [50] and evolved log titers along the phylogeny corresponding to the largest non-recombinant partition of each alignment according to Brownian motion. We applied our method to each of the 10 simulated datasets ignoring recombination and did not observe any false positives, suggesting that our results are robust to recombination at this level.

A single, maximum likelihood phylogeny for the panel of viruses was inferred with PhyML [51] by specifying a general time reversible model of nucleotide substitution and an estimated proportion of invariable sites. Site-to-site variation in the evolutionary rate of the variable sites was modeled by a four-category discrete gamma distribution with unit mean. Subtree pruning and regrafting and nearest neighbor interchange were used to search the tree space for the optimal topology, starting with a BioNJ tree and five random trees. The root was identified by including an outgroup of SIV and HIV-1 groups N, O and P reference sequences obtained from the LANL HIV sequence database.

### Evolutionary models

Our computational approach to identifying sites targeted by broadly neutralizing antibodies is an extension of the method of Lacerda et al. [42], which was originally developed to predict T cell epitopes by identifying escape sites where viral evolution correlated with the immune type of the host. Here, we did not have information on the host conditions under which each of the panel viruses evolved and therefore could not infer immune escape *per se*. Instead, we identified amino acid positions in HIV-1 envelope that influenced sensitivity to antibody neutralization across a phylogeny. This was achieved by specifying an evolutionary model that allowed the amino acid equilibrium frequencies at each site to depend on neutralization titer. To avoid estimating 19 frequency parameters at each site, we selected a neutralization-sensitive virus, which we refer to as the “reference sequence,” and modeled only the equilibrium frequency of the amino acid present in this sequence at each site. Reference sequences were selected on the basis of high neutralization titers (which varied depending on the potency of each serum sample) and the availability of cloned envelopes for later validation studies. Where possible, the early autologous virus (isolated from the individual from whom the broadly neutralizing serum was obtained) was also used, as most of these viruses are known to be sensitive to the broadly neutralizing antibodies that develop later. The reference sequences used for each serum and their Genbank accession numbers are provided in Additional file 2: Table S1.

We adapted the evolutionary model of Halpern and Bruno [52] in which the instantaneous rate of codon substitution *q*^*k*^_*ij*_ at site *k* is parameterized as the product of the mutation rate *μ*_*ij*_ and the probability of fixation *f*^*k*^_*ij*_ relative to that of a neutral mutation; that is, *q*^*k*^_*ij*_ ∝ *μ*_*ij*_ × *f*^*k*^_*ij*_ / (1/*N*) for codons *i* and *j* and effective haploid population size *N*. Assuming a time-reversible model of codon substitution, the authors showed that the fixation probability of a mutation from codon *i* to codon *j* with relative selective advantage 1 + *s*, *s* ≪ 1, can be written as

(1) $$f_{\mathrm{ij}}^{k}=N^{-1}\frac{\text{ln}\left(\frac{\nu_{j}^{k}\mu_{\mathrm{ji}}}{\nu_{i}^{k}\mu_{\mathrm{ij}}}\right)}{1-\frac{\nu_{i}^{k}\mu_{\mathrm{ij}}}{\nu_{j}^{k}\mu_{\mathrm{ji}}}},$$

where $\nu_{j}^{k}$ is the equilibrium frequency of codon *j* at site *k*.

We parameterized the site-specific equilibrium frequencies as

(2) $$\nu_{j}^{k}=\left\{\begin{array}{c} \gamma_{k}\;\times \frac{\pi_{j}}{\pi_{\Gamma }}\;\mathrm{if}\;j\in \Gamma \\ \left(1-\gamma_{k}\right)\;\times \frac{\pi_{j}}{1-\pi_{\Gamma }}\;\mathrm{if}\;j\notin \Gamma , \end{array}\right)$$

where *γ*_*k*_ is the equilibrium frequency of the reference amino acid at site *k*, *π*_*j*_ is the equilibrium frequency of codon *j* in the absence of selection, Γ represents the set of codons that encode the reference amino acid and *π*_*Γ*_ = ∑ _*i* ∈ *Γ*_*π*_*i*_. The factor involving the *π*_*j*_ terms distributes the reference amino acid frequency among the codons that encode it in such a way as to maintain the codon usage bias observed over the entire alignment.

We used an HKY85 model for the mutation rate *μ*_*ij*_, with the nucleotide equilibrium frequencies estimated empirically from the full alignment. Because we fitted this model to coding sequences, our estimates will not only reflect the mutational process, but will also capture selection induced through the genetic code. We do not expect that our model results will be sensitive to this misspecification. Ideally, the mutation parameters should be estimated from non-coding nucleotide sequences [52]. The estimated nucleotide frequencies from the mutation model were used to construct F1 × 4 estimates of the codon frequencies *π*_*j*_ expected in the absence of selection.

Assuming a time-reversible mutation process, substituting (2) into (1) yields *f*^*k*^_*ij*_ = 1/*N*, and hence *s* = 0, for all mutations that do not involve a substitution of the reference residue for or by another amino acid. This formulation therefore permits only directional selection that alters the codon equilibrium frequencies $\nu_{j}^{k}$ based on the frequency *γ*_*k*_ of the reference amino acid at site *k*. Nonsynonymous mutations that do not involve the reference amino acid would have the same fixation probability (1/*N*) as synonymous mutations and would therefore be modeled as selectively neutral. However, nonsynonymous mutations at different sites are likely to evolve under purifying and diversifying selection that alters the fixation probabilities without affecting codon frequencies. To accommodate this in our model, we parameterize the fixation probability of a nonsynonymous mutation at site *k* as *ω*_*k*_ × 1/*N*. A value of *ω*_*k*_ = 1 indicates that nonsynonymous and synonymous mutations are fixed with the same probability at site *k* in the absence of directional selection. Values of *ω*_*k*_ < 1 and *ω*_*k*_ > 1 imply purifying and diversifying selection at site *k*, respectively. The codon substitution rates are then defined as

(3) qijk∝μijifsynonymousμijωkifnonsynonymous,i∉Γ,j∉ΓμijωkInγk1-γkπΓ1-πΓ1-πΓ1-πΓγk1-γkifnonsynonymous,i∉Γ,j∈ΓμijωkIn1-γkγk1-πΓπΓ1-1-πΓπΓ1-γkγkifnonsynonymous,i∈Γ,j∉Γ0ifiandjdifferat>1ntposition.

A similar codon model that distinguishes between diversifying and directional selection was recently considered by Murrell et al. [53].

To identify codon sites associated with sensitivity to antibodies, we allowed the equilibrium frequency *γ*_*k*_ of the reference amino acid to depend on neutralization titers. More specifically, let *γ*_*0k*_ be the equilibrium frequency of the reference amino acid at site *k* among sensitive (high ID_50_ titer) viruses and let *γ*_*1k*_ denote this frequency for resistant (low ID_50_ titer) viruses. We set *γ*_*k*_ = *p γ*_*0k*_ + (1 – *p*) *γ*_*1k*_, where *p* ∈ [0,1] is a measure of neutralization sensitivity that is monotonically increasing in ID_50_ neutralization titer. Since the frequency of the reference residue among sensitive viruses was expected to be at least as large as that among resistant viruses, we constrained *γ*_*0k*_ ≥ *γ*_*1k*_ where equality implied that a site was not associated with antibody neutralization.

For the model defined in Equation (3), nonsynonymous substitutions toward the reference amino acid occur at a higher rate if the relative equilibrium frequency of the reference residue *γ*_*k*_/(1-*γ*_*k*_) is larger than its expected value in the absence of directional selection, *π*_*Γ*_/(1-*π*_*Γ*_). Substitutions in the opposite direction are favored if the converse is true. Thus, a neutralization-resistant virus (*p* close to 0) would be more likely to substitute the reference amino acid for another residue at sites where *γ*_*0k*_ > *γ*_*1k*_. Similarly, substitutions in favor of the reference residue will be more likely at these sites among neutralization-sensitive viruses (*p* close to 1). This pattern of evolution would reduce the prevalence of the reference residue among resistant viruses and increase its frequency among sensitive viruses, consistent with our expectation of sites associated with antibody neutralization.

The value of *p* along each branch of the phylogeny was determined by the neutralization titer of the sequence at the tip of that branch. This parameter may be specified by selecting appropriate titer thresholds and setting *p* = 0 if the titer is high (sensitive), *p* = 1 if the titer is low (resistant) and setting *p* equal to the proportion of classified viruses that were sensitive when the titer is an intermediate value or unknown. The method of Gnanakaran et al. [15] classifies viruses as neutralization sensitive or resistant by applying such a threshold. Here, ID_50_ titer was treated as a continuous variable and mapped onto the [0,1] scale to obtain values for *p*. Due to the skewness of the ID_50_ titer distribution, we defined *p* = [*t* – min(*t*)]/max[*t* – min(*t*)], where *t* is the natural logarithm of titer. When the titer was unknown, such as at all ancestral nodes, we set *p* equal to its median value among all sequences with observed titers. We also considered imputing the unknown titer values using the method of Felsenstein [45] (see Discussion).

Although Equation (3) defines a time-reversible model along each branch of the phylogeny, the process is not time reversible when considered over the entire tree. Consequently, the location of the root node and initial codon frequencies must be specified for valid likelihood-based inference. This specification is trivial when the median titer is used to define *p* at all nodes with unknown titers, since any interior node that is not the immediate ancestor of a leaf node can then act as the root [42]. However, identification of a specific node as the root is required when the imputed titer values differ between ancestral nodes, such as with Felsenstein’s method of reconstruction [45].

Given an inferred topology and F1 × 4 codon equilibrium frequencies, all model parameters, except *γ*_*0k*_ and *γ*_*1k*_, were estimated by fitting a GY94 × HKY85 model in HyPhy [54] with *ω*_*k*_ drawn from a three-category general discrete distribution. Fixing the set, *θ*, of these parameters at their estimated values, the probability *p*(*x*_*k*_| *θ*, *γ*_*0k*_, *γ*_*1k*_) of the codon data *x*_*k*_ at site *k* can be computed under the model in Equation (3) for any pair (*γ*_*0k*_, *γ*_*1k*_): 0 ≤ *γ*_*1k*_ ≤ *γ*_*0k*_ ≤ 1 using Felsenstein’s pruning algorithm. To compute the likelihood of our epitope model, we treated *γ*_*0k*_ and *γ*_*1k*_ as nuisance parameters with a flat joint distribution and integrated them out of the likelihood function using the adaptive numerical integration routine available in the cubature R package. We also computed the likelihood of a non-epitope model that does not allow for an association between an alignment site and neutralization sensitivities by setting *γ*_*0k*_ = *γ*_*1k*_ = *γ*_*k*_ and integrating *γ*_*k*_ out of the likelihood function with *γ*_*k*_ ~ *U*(0,1).

The data-based evidence in support of the epitope model relative to the non-epitope model at site *k* was then assessed using the scaled Bayes factor

$$B_{k}=2\text{ln}\left(\frac{L_{E}\left(\theta |x_{k}\right)}{L_{N}\left(\theta |x_{k}\right)}\right),$$

where *L*_*E*_(*θ* | *x*_*k*_) and *L*_*N*_(*θ* | *x*_*k*_) are the integrated likelihoods under the epitope and non-epitope models, respectively. Kass and Raftery [55] suggest that a value of 2 < *B*_*k*_ < 6 can be interpreted as positive, but weak evidence against the null model, while *B*_*k*_ ≥ 6 indicates strong evidence against the null model. We used the latter criterion to identify sites for experimental validation.

By performing model comparisons at each of 818 amino acid positions per serum, we anticipated that some sites with *B*_*k*_ ≥ 6 would be false positives. To address this issue, we computed the local false discovery rate associated with each of our scaled Bayes factors. The local false discovery rate (LFDR) is defined as the probability of the null (non-epitope) model given the codon data *x*_*k*_ at site *k*[56]. For each site identified as significantly associated with neutralization titer, the LFDR indicates the probability that the site has been classified incorrectly, given the data *x*_*k*_ and the prior probability *π*_0_ of the null model. The LFDR for site *k* may be computed by observing that

$$\frac{1-{\text{LFDR}}_{k}}{{\text{LFDR}}_{k}}=\underset{\text{posterior}\;\text{odds}}{\underbrace{\frac{p_{E}\left(\theta |x_{k}\right)}{p_{N}\left(\theta |x_{k}\right)}}}=\underset{\text{Bayes}\;\text{factor}}{\underbrace{\frac{L_{E}\left(\theta |x_{k}\right)}{L_{N}\left(\theta |x_{k}\right)}}}\;\cdot \;\underset{\text{prior}\;\text{odds}}{\underbrace{\frac{1-\pi_{0}}{\pi_{0}}}}$$

and rearranging terms to obtain

(4) $${\text{LFDR}}_{k}=\frac{\frac{\pi_{0}}{1-\pi_{0}}}{\text{exp}\left(\frac{B_{k}}{2}\right)+\frac{\pi_{0}}{1-\pi_{0}}}$$

Use of Equation (4) required an estimate of *π*_0_ which we obtained from the following relation

(5) $$p\left(B_{k}>0\right)=p_{N}\left(B_{k}>0\right)\pi_{0}+p_{E}\left(B_{k}>0\right)\left(1-\pi_{0}\right),$$

where *p*_*N*_(*B*_*k*_ > 0) and *p*_*E*_(*B*_*k*_ > 0) denoted the probability of a positive scaled Bayes factor if the data was generated under the non-epitope and epitope models, respectively. The probability *p*(*B*_*k*_ > 0) can be approximated as the proportion of observed scaled Bayes factors greater than zero for any particular serum sample. To approximate *p*_*N*_(*B*_*k*_ > 0), we permuted the assignment of ID_50_ titers to the pseudoviruses and recorded the proportion of scaled Bayes factors greater than zero. In this respect, we considered only sites at which the frequency of the most prevalent amino acid was <95%, since invariant sites do not provide evidence for either model. The median proportion over 100 such permutations was used as an estimate of *p*_*N*_(*B*_*k*_ > 0). (The mean proportion was not substantively different.) These estimates are reported in Additional file 2: Table S2. For a suitable choice of *p*_*E*_(*B*_*k*_ > 0), *π*_0_ may be computed from Equation (5) and used to obtain the LFDR for a given Bayes factor. We set *p*_*E*_(*B*_*k*_ > 0) = 1 to obtain an upper bound on the LFDR. Our reported false discovery rates should therefore be regarded as conservative.

### Conformational epitope prediction

The method described above can identify individual sites associated with neutralization titer. A set of such predictions in close proximity on the primary sequence provides evidence for a linear B cell epitope. Similarly, a set of predictions clustered on the three-dimensional structure provides evidence for a conformational epitope. For any set of amino acids, *C*, the likelihood that these sites all support the epitope model and that all other sites are generated under the non-epitope model may be computed as

$$L\left(C\right)=\prod_{k\in C}L_{E}\left(\theta |x_{k}\right)\prod_{k\notin C}L_{N}\left(\theta |x_{k}\right)$$

We introduced a simple spherical model of an epitope that can identify sets of sites that support the epitope model and are clustered in three-dimensional space. A Metropolis algorithm was used to explore the posterior density of the location and size of the sphere (see [57] for a general description of the Metropolis algorithm). The posterior probability that any three-dimensional location lies within the conformational epitope was then computed as the proportion of times that the location falls within the sampled spheres.

We implemented the Metropolis algorithm in Mathematica 8, using the 2B4C Protein Data Bank structure for gp120 and the likelihoods computed for each site with HyPhy [54]. We assumed a uniform prior on the three-dimensional coordinate vector defining the centre of the sphere and a gamma prior, *π*(*r*), on the radius *r* of the sphere with a shape parameter of 2 Å and a scale parameter of 4 Å (mean of 8 Å). At the outset, a sphere was generated by randomly selecting an amino acid as the centre of the sphere and drawing a random radius from its prior distribution. A new sphere was proposed by independently drawing each of the three coordinates and the radius from a normal distribution centered on the parameter value of the current sphere with a fixed standard deviation of 3 Å. A move from the current sphere *C* with radius *r* to a new sphere *C’* with a radius *r’* located elsewhere in the protein was accepted with probability

$$\alpha =\text{min}\left(\frac{L\left(C'\right)\pi \left(r'\right)}{L\left(C\right)\pi \left(r\right)},1\right).$$

A subsequent sphere was then proposed as described above and the process repeated until the Markov chain converged to its stationary distribution. We used a burn-in period of 10 000 iterations, after which spheres were sampled from their posterior distribution at every 100th iteration for 500 000 iterations.

### Identification of coevolving sites

We used a modified version of the evolutionary network model of Poon et al. [38] to identify co-evolving sites. The evolutionary events that generated the observed sequence alignment were reconstructed by maximum likelihood under an MG94 × GTR model of codon evolution. For each of the terminal branches, the codon sites that were inferred to have undergone nonsynonymous substitutions were encoded with a 1 and other codon sites were encoded with a 0. This produced an augmented dataset with each terminal branch corresponding to an independent observational vector of the substitution events that occurred along that branch at each codon site and the neutralization titer observed at the tip of the branch. These data were used to construct a Bayesian network under the assumption of log-normal titers. In this context, a Bayesian network is a representation of the joint probability distribution over neutralization titer and the substitution events at all codon positions. The network may be visualized as a graph with nodes representing variables (codon sites and neutralization titer) and edges indicating dependencies between the variables. The evidence in support of each relationship is quantified with a posterior probability. Importantly, by considering the complete distribution of the data, a Bayesian graphical model is able to resolve conditional dependencies, where one variable is related to another only through a mutual relationship with a third variable. The model was implemented in HyPhy with an experimental batch file provided by Dr AFY Poon.

### Phylogenetically-corrected Fisher’s exact tests

We compared the predictions of our evolutionary model to those of the signature detection method of Gnanakaran et al. [15]. Briefly, this method uses phylogenetically-corrected Fisher’s exact tests to identify significant associations between amino acid changes along the terminal branches of the viral phylogeny and a binary classification of neutralization phenotypes at the tips of the branches. Three neutralization classification schemes were considered; viruses were classified as neutralization resistant if their ID_50_ titers were less than the first quartile, less than the median or less than the third quartile. Ancestral sequences were reconstructed by maximum likelihood using a general time reversible model of nucleotide evolution with site-specific rate heterogeneity. For each possible ancestral amino acid at each site, a contingency table was constructed to test whether the proportion of neutralization sensitive viruses was significantly different between extant viruses that contained the ancestral amino acid at the site versus those that did not. Due to the large number of tests performed, *q*-values were used to judge statistical significance while controlling the false positive rate. All sites with at least one *q* ≤ 1/3 (twice as likely to be a true positive as a false positive) were considered as worthy of further investigation. Only the smallest *q*-value obtained for each site is reported in Additional file 2: Table S3.

### Experimental validation

A subset of the residues predicted to be associated with neutralization sensitivity were tested for their effect on neutralization sensitivity in one to three sensitive envelope backbones per serum sample using site-directed mutagenesis. Residues of interest were mutated to alanine in all cases except for position 169, which was mutated to glutamic acid as described previously [34]. Mutagenesis was performed using the Stratagene QuickChange II kit (Stratagene) and confirmed by sequence analysis. Envelope-pseudotyped viruses from mutated and parental envelope clones were obtained by co-transfecting the envelope plasmid with pSG3ΔEnv using Fugene transfection reagent (Roche) into 293 T cells as previously described [7]. Neutralization was measured by a reduction in luciferase gene expression after single round infection of JC53bl-13 cells with envelope-pseudotyped viruses [7]. Titers were calculated as the reciprocal plasma dilution (ID_50_) causing 50% reduction of relative light units. The effect of mutations on neutralization sensitivity was calculated as a fold change in neutralization titers of the mutant virus compared to the unmutated parental clone. A model prediction was regarded as validated if a mutation at the corresponding site produced at least a twofold reduction in ID_50_ titer in at least one backbone.

### Software availability

The likelihoods of the evolutionary models were computed on a computer cluster using the parallel processing capabilities of HyPhy and the R Language and Environment for Statistical Computing. We are currently working on an online tool to implement this methodology. All computer code is freely available from the corresponding author.

### Availability of supporting data

The data set supporting the results of this article is provided in Additional file 3: Dataset S1.

## Competing interests

The authors declare that they have no competing interests.

## Authors’ contributions

CW and SSAK conceived, implemented and led the CAPRISA 002 Acute Infection study. LM, CW and CS conceived and designed the experiments. PLM, ESG, MN, MM, CKW, DS, MS, RTB and JM performed the experiments. ML, NKN, BM, BTMK and MK analyzed data. SSAK, HG ad KG contributed reagents and materials. ML, PLM, LM, CW and CS wrote the manuscript. All authors read and approved the final manuscript.

## Supplementary Material

Additional file 1: Figure S1 Model predictions for CAP256. Scaled Bayes factors using the (A) autologous CAP256, (B) CAP210 and (C) CAP45 reference sequences. (D) Bayesian evolutionary-network model. (E) Posterior probabilities of a conformational epitope using the three-dimensional model with a ConC reference sequence. The posterior probabilities are shaded as described in the legend for Figure 4B. There is some evidence of a conformational epitope in the V3 region (posterior probabilities as high as 0.367). **Figure S2.** Model predictions for CAP8. Scaled Bayes factors using the (A) ConC, (B) Q23 and (C) TRO reference sequences. (D) Bayesian evolutionary-network model. (E) Posterior probabilities of a conformational. **Figure S3.** Model predictions for CAP257. Scaled Bayes factors using the (A) ConC and (B) Q842 reference sequences. (C) Bayesian evolutionary-network model. (D) Posterior probabilities of a conformational epitope using the three-dimensional model with a ConC reference sequence. The posterior probabilities are shaded as described in the legend for Figure 4B. There is some evidence of a conformational epitope in the V3 region (posterior probabilities as high as 0.361). **Figure S4.** Model predictions for CAP255. Scaled Bayes factors using the (A) autologous CAP255, (B) TRO and (C) Q23 reference sequences. **Figure S5.** Model predictions for CAP177. Scaled Bayes factors using the (A) ConC, (B) Q23 and (C) TRO reference sequences. (D) Bayesian evolutionary-network model. **Figure S6.** Model predictions for CAP206. Scaled Bayes factors using the (A) ZM197, (B) autologous CAP206, (C) CAP45, (D) Q23, (E) COT6 and (F) TRO reference sequences. (G) Bayesian evolutionary-network model. **Figure S7.** Model predictions for CAP248. Scaled Bayes factors using the (A) ConC, (B) CAP45 and (C) DU156 reference sequences. (D) Bayesian evolutionary-network model. **Figure S8.** Scaled Bayes factors for the CAP256 serum obtained after imputing ancestral titers with the median observed titer and titers reconstructed with Felsenstein’s method. Click here for file

Additional file 2: Table S1 Reference sequences. **Table S2.** Estimates used to compute LFDRs. **Table S3.** Significant associations obtained with the method of Gnanakaran et al. [15]. **Table S4.** Sites with scaled Bayes factors ≥ 6 using reconstructed titers. Click here for file

Additional file 3: Dataset S1 HIV-1 gp160 alignment. Click here for file

Additional file 4: Dataset S2 Genbank accession numbers and neutralization titers for the virus panel. Click here for file
