# Supplementary material for: Identification of broadly neutralizing antibody epitopes in the HIV-1 envelope glycoprotein using evolutionary models
Source: Virol J. 2013 Dec 2;10:347. doi: 10.1186/1743-422X-10-347 (PMC4220805; doi:10.1186/1743-422X-10-347)
Supplement: Additional file 1: Figure S1 — Model predictions for CAP256. Scaled Bayes factors using the (A) autologous CAP256, (B) CAP210 and (C) CAP45 reference sequences. (D) Bayesian evolutionary-network model. (E) Posterior probabilities of a conformational epitope using the three-dimensional model with a ConC reference sequence. The posterior probabilities are shaded as described in the legend for Figure 4B. There is some evidence of a conformational epitope in the V3 region (posterior probabilities as high as 0.367). Figure S2. Model predictions for CAP8. Scaled Bayes factors using the (A) ConC, (B) Q23 and (C) TRO reference sequences. (D) Bayesian evolutionary-network model. (E) Posterior probabilities of a conformational. Figure S3. Model predictions for CAP257. Scaled Bayes factors using the (A) ConC and (B) Q842 reference sequences. (C) Bayesian evolutionary-network model. (D) Posterior probabilities of a conformational epitope using the three-dimensional model with a ConC reference sequence. The posterior probabilities are shaded as described in the legend for Figure 4B. There is some evidence of a conformational epitope in the V3 region (posterior probabilities as high as 0.361). Figure S4. Model predictions for CAP255. Scaled Bayes factors using the (A) autologous CAP255, (B) TRO and (C) Q23 reference sequences. Figure S5. Model predictions for CAP177. Scaled Bayes factors using the (A) ConC, (B) Q23 and (C) TRO reference sequences. (D) Bayesian evolutionary-network model. Figure S6. Model predictions for CAP206. Scaled Bayes factors using the (A) ZM197, (B) autologous CAP206, (C) CAP45, (D) Q23, (E) COT6 and (F) TRO reference sequences. (G) Bayesian evolutionary-network model. Figure S7. Model predictions for CAP248. Scaled Bayes factors using the (A) ConC, (B) CAP45 and (C) DU156 reference sequences. (D) Bayesian evolutionary-network model. Figure S8. Scaled Bayes factors for the CAP256 serum obtained after imputing ancestral titers with the median observed titer and titers reconstructed with [file 1743-422X-10-347-S1.pdf]

Figure S1

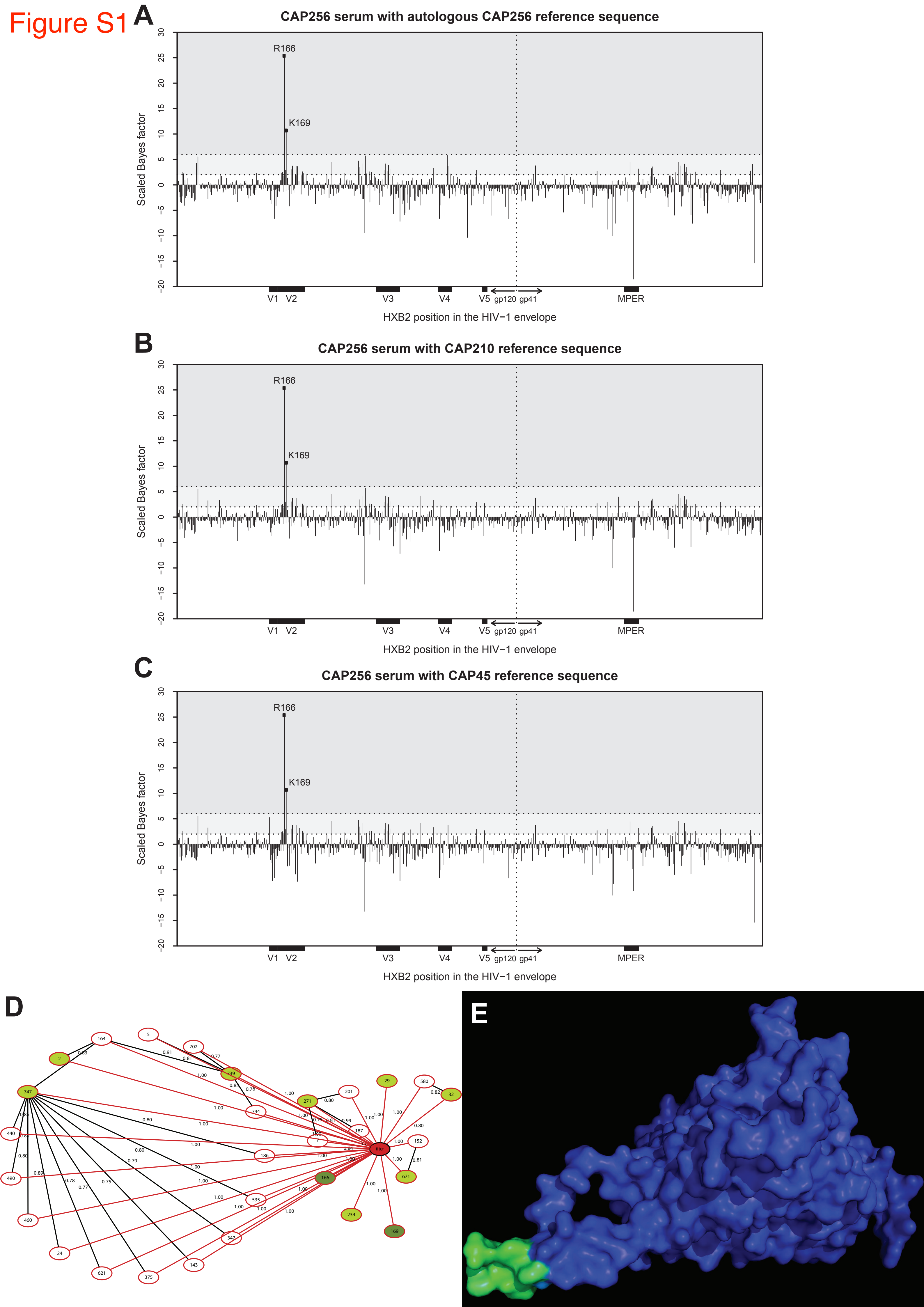
A
CAP256 serum with autologous CAP256 reference sequence
Scaled Bayes factor
R166
K169
V1
V2
V3
V4
V5
gp120
gp41
MPER
HXB2 position in the HIV−1 envelope
B
CAP256 serum with CAP210 reference sequence
Scaled Bayes factor
R166
K169
V1
V2
V3
V4
V5
gp120
gp41
MPER
HXB2 position in the HIV−1 envelope
C
CAP256 serum with CAP45 reference sequence
Scaled Bayes factor
R166
K169
V1
V2
V3
V4
V5
gp120
gp41
MPER
HXB2 position in the HIV−1 envelope
D
titer
E

Figure S2

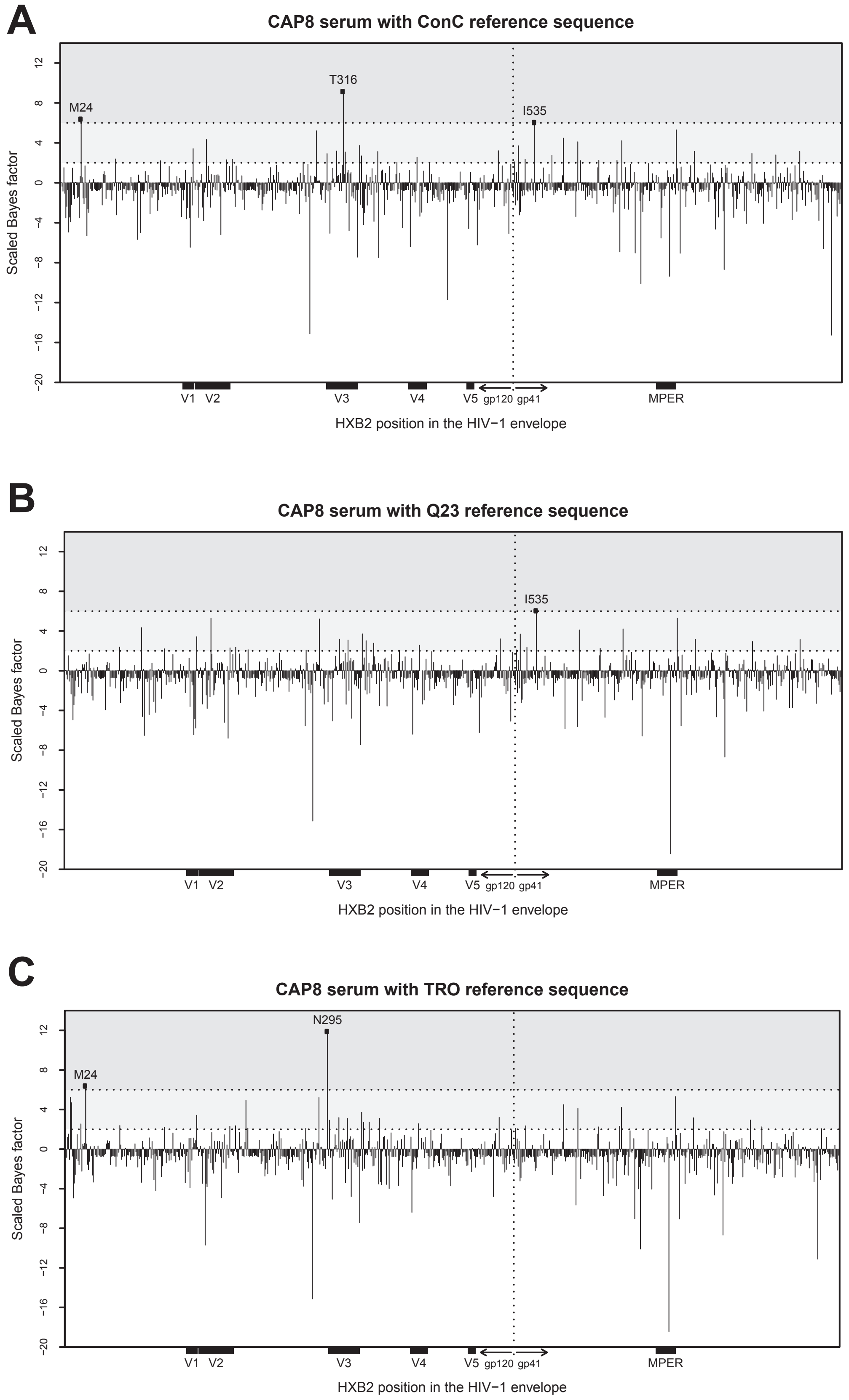
A
CAP8 serum with ConC reference sequence
Scaled Bayes factor
M24
T316
I535
V1 V2 V3 V4 V5 gp120 gp41 MPER
HXB2 position in the HIV−1 envelope
B
CAP8 serum with Q23 reference sequence
Scaled Bayes factor
I535
V1 V2 V3 V4 V5 gp120 gp41 MPER
HXB2 position in the HIV−1 envelope
C
CAP8 serum with TRO reference sequence
Scaled Bayes factor
N295
M24
V1 V2 V3 V4 V5 gp120 gp41 MPER
HXB2 position in the HIV−1 envelope

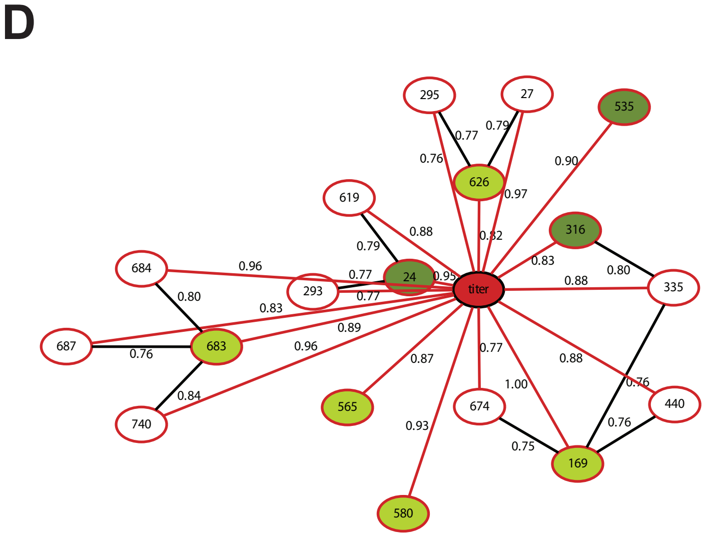
D
titer
295
27
535
626
619
316
684
293
24
335
687
683
740
565
674
440
169
580

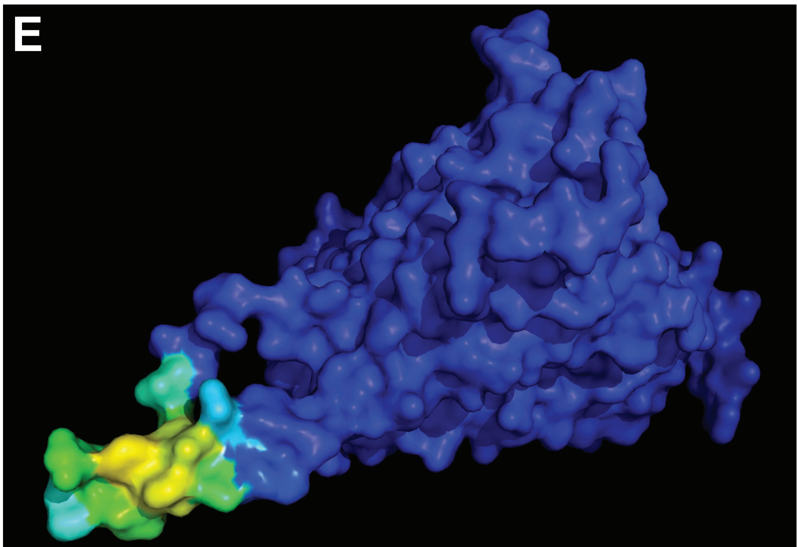
E

Figure S3

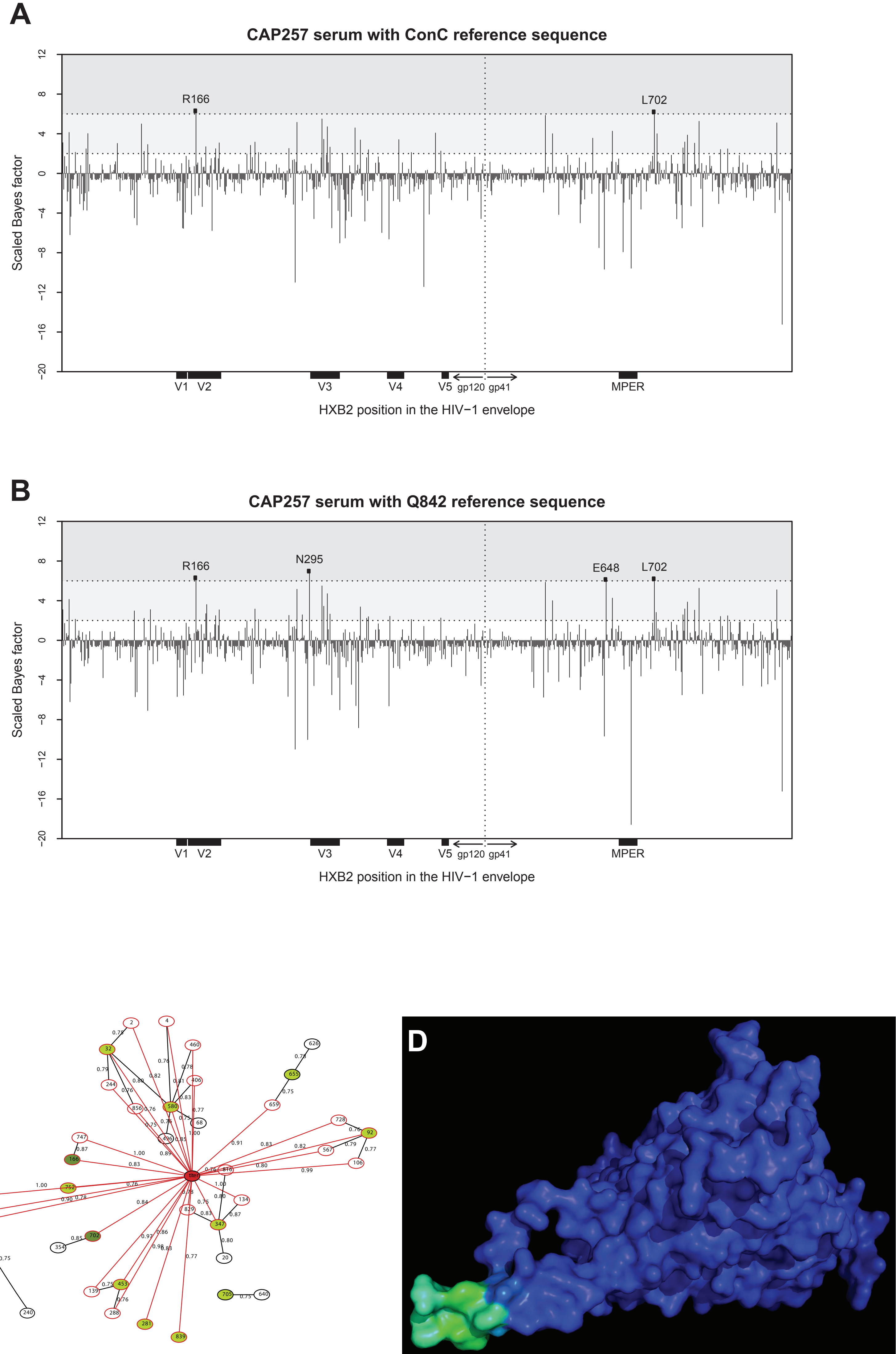

A
CAP257 serum with ConC reference sequence
Scaled Bayes factor
12
8
4
0
−4
−8
−12
−16
−20
R166
L702
V1
V2
V3
V4
V5
gp120
gp41
MPER
HXB2 position in the HIV−1 envelope
B
CAP257 serum with Q842 reference sequence
Scaled Bayes factor
12
8
4
0
−4
−8
−12
−16
−20
R166
N295
E648
L702
V1
V2
V3
V4
V5
gp120
gp41
MPER
HXB2 position in the HIV−1 envelope
C
D

Figure S4

A

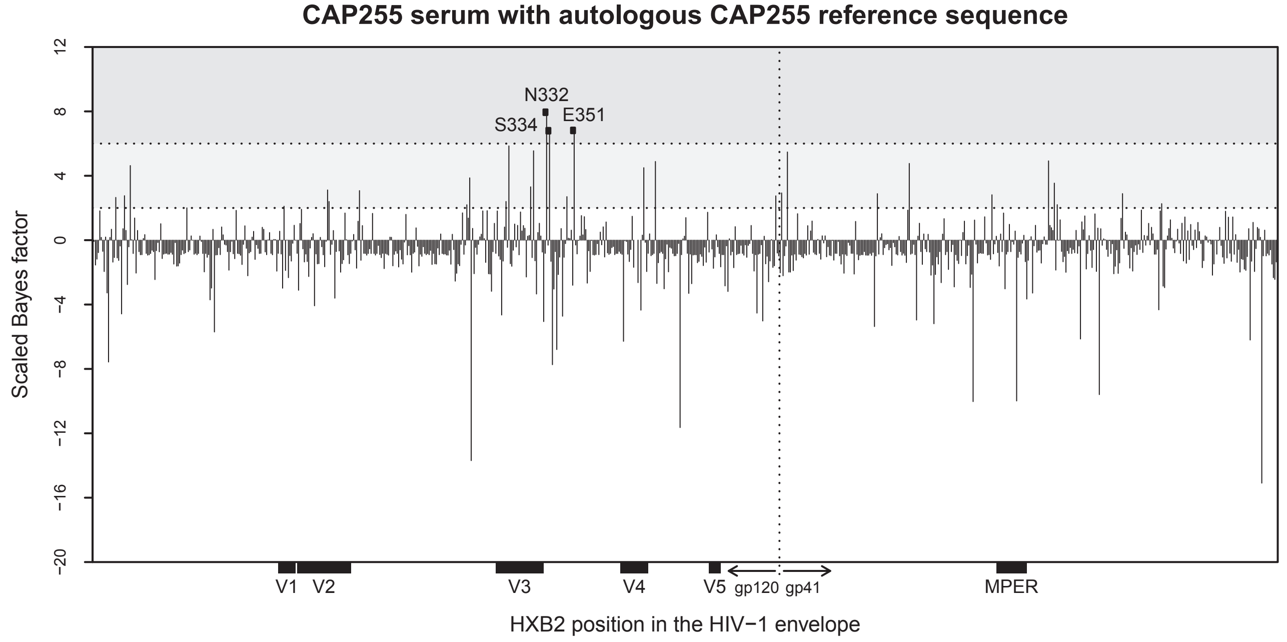

B

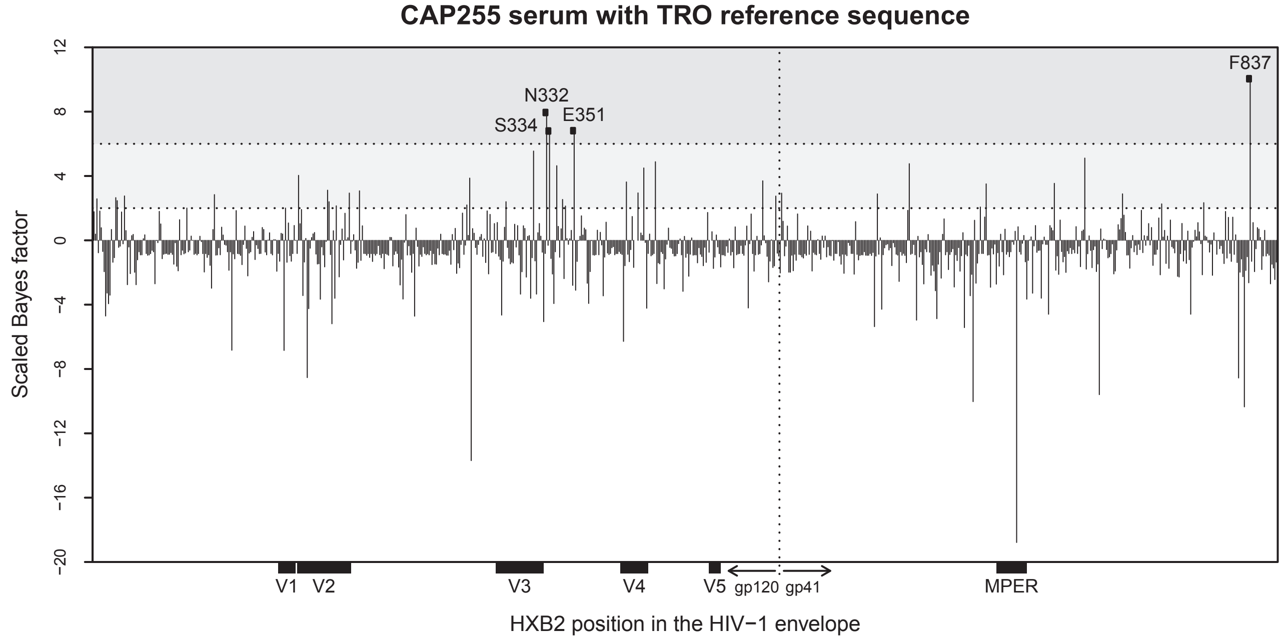

C

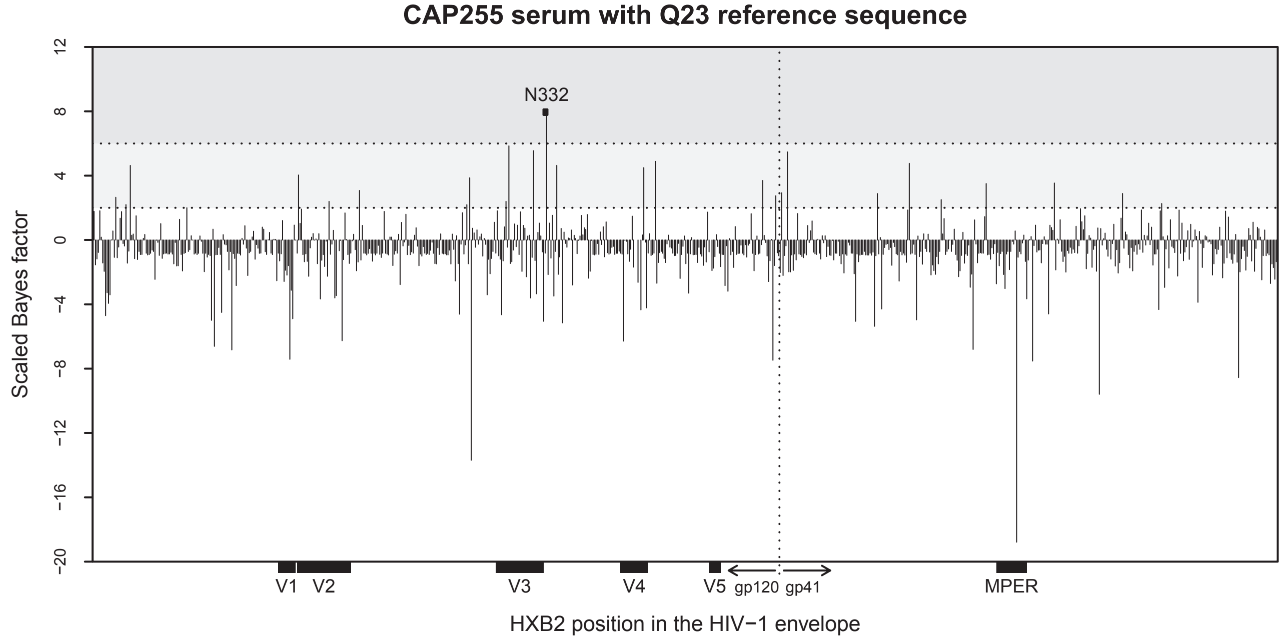

Figure S5

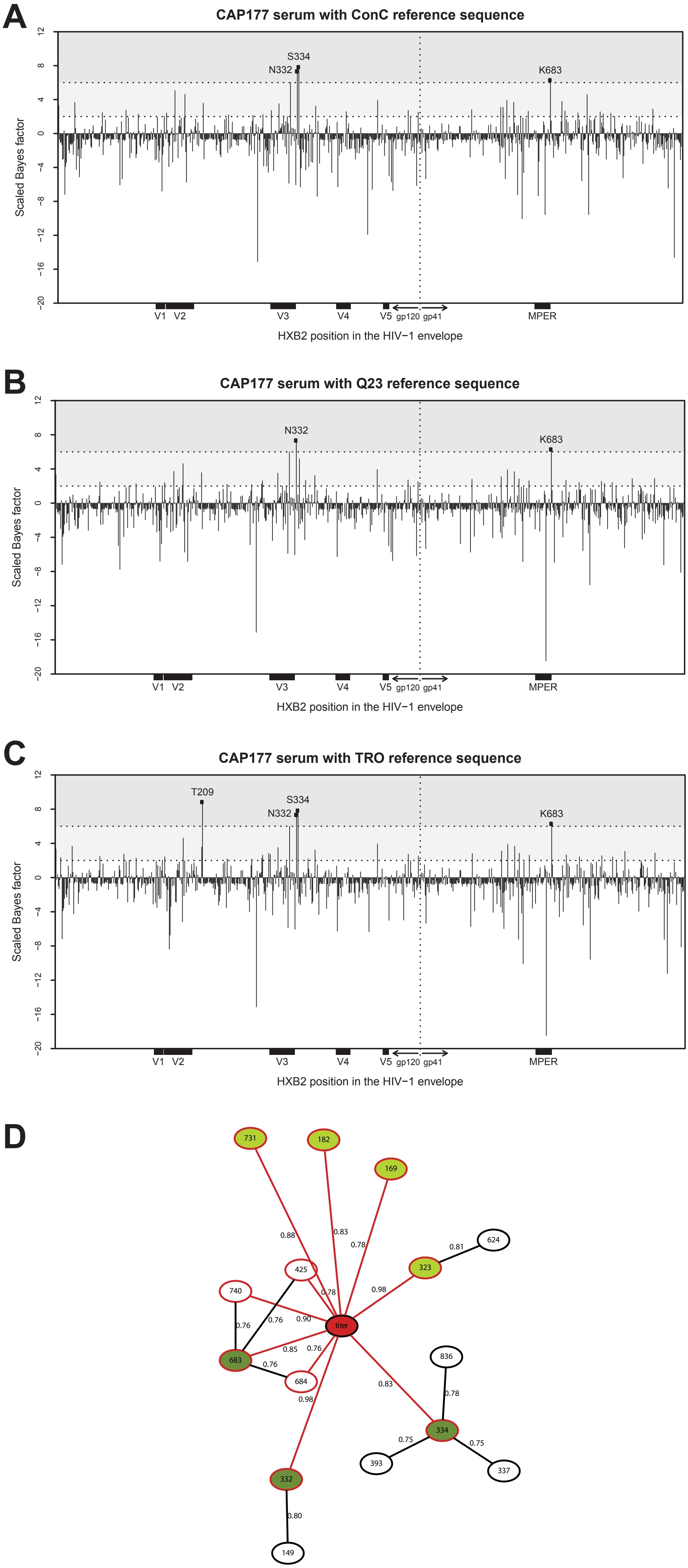
A
CAP177 serum with ConC reference sequence
Scaled Bayes factor
12
8
4
0
−4
−8
−12
−16
−20
S334
N332
K683
V1
V2
V3
V4
V5
gp120
gp41
MPER
HXB2 position in the HIV−1 envelope
B
CAP177 serum with Q23 reference sequence
Scaled Bayes factor
N332
K683
V1
V2
V3
V4
V5
gp120
gp41
MPER
HXB2 position in the HIV−1 envelope
C
CAP177 serum with TRO reference sequence
Scaled Bayes factor
T209
S334
N332
K683
V1
V2
V3
V4
V5
gp120
gp41
MPER
HXB2 position in the HIV−1 envelope
D
731
182
169
624
323
425
740
titer
683
684
836
334
393
337
332
149
0.88
0.83
0.78
0.81
0.98
0.78
0.90
0.76
0.76
0.85
0.76
0.76
0.98
0.83
0.78
0.75
0.75
0.80

A

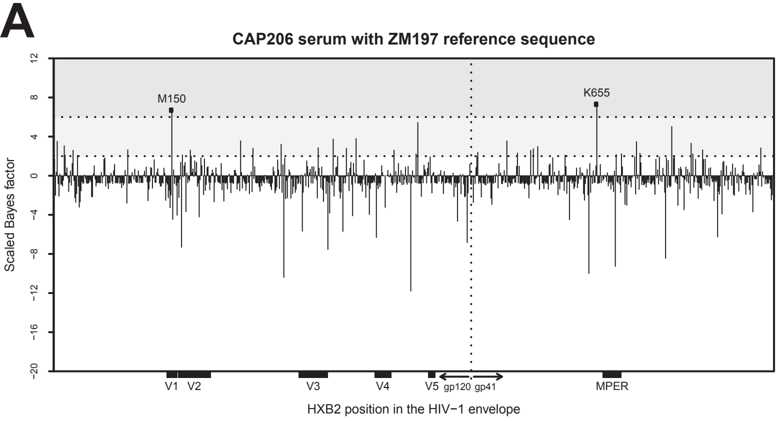

D

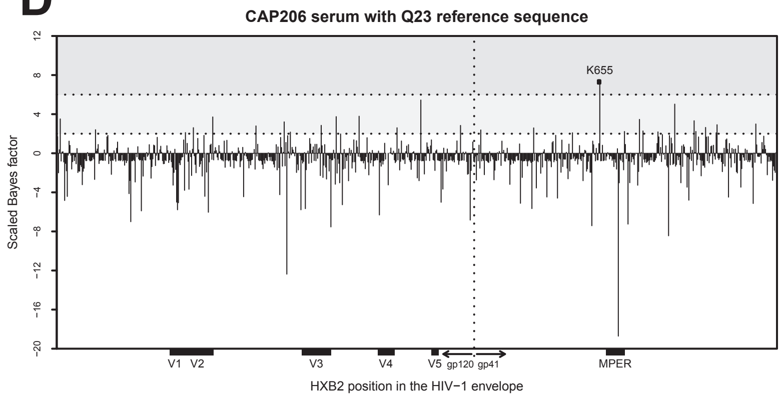

Figure S6

B

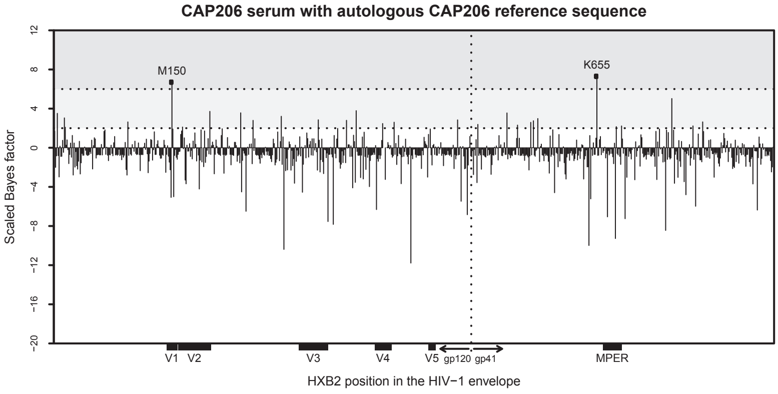

E

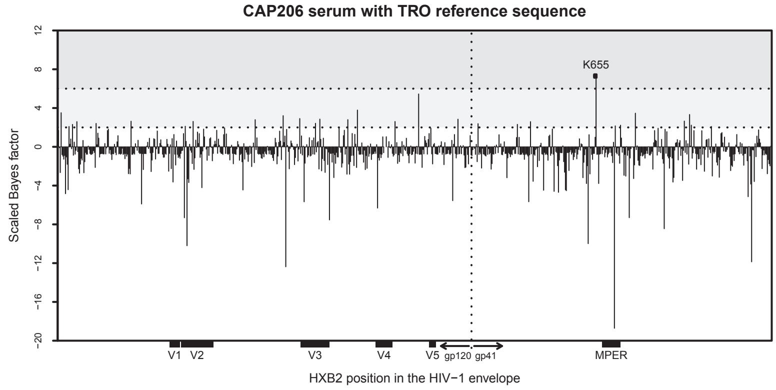

C

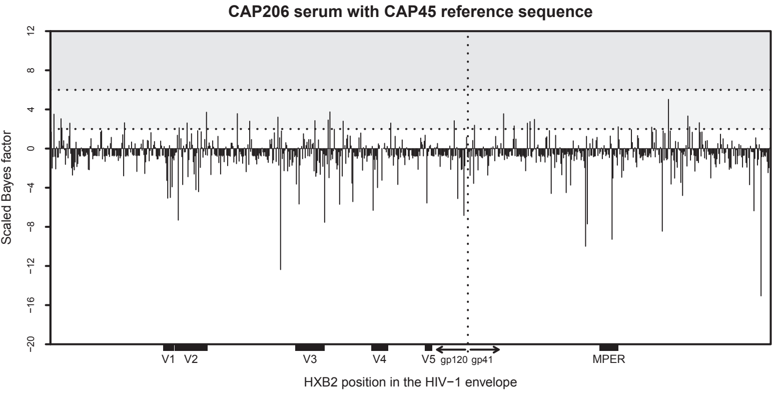

F

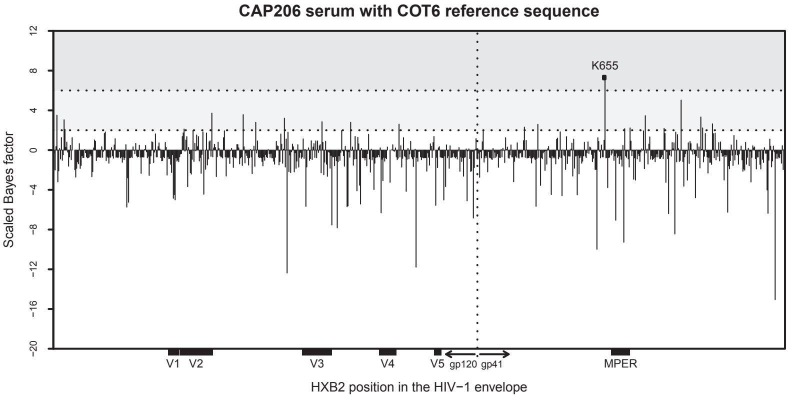

G

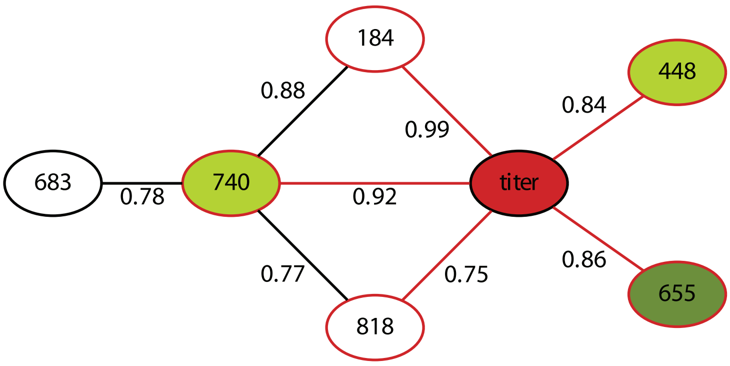

Figure S7

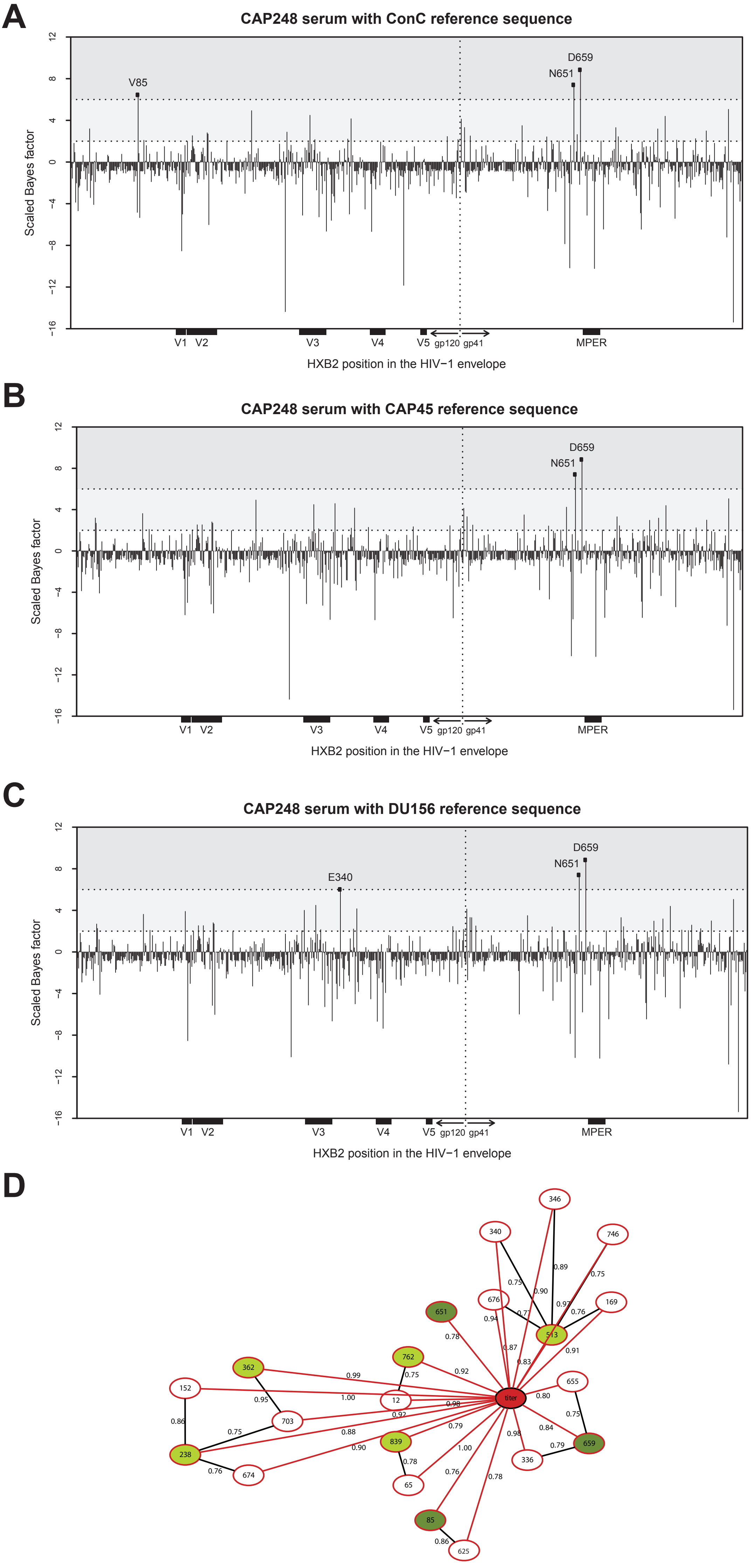
A
CAP248 serum with ConC reference sequence
Scaled Bayes factor
V85
N651
D659
V1 V2 V3 V4 V5 gp120 gp41 MPER
HXB2 position in the HIV−1 envelope
B
CAP248 serum with CAP45 reference sequence
Scaled Bayes factor
N651
D659
V1 V2 V3 V4 V5 gp120 gp41 MPER
HXB2 position in the HIV−1 envelope
C
CAP248 serum with DU156 reference sequence
Scaled Bayes factor
E340
N651
D659
V1 V2 V3 V4 V5 gp120 gp41 MPER
HXB2 position in the HIV−1 envelope
D
titer

Figure S8

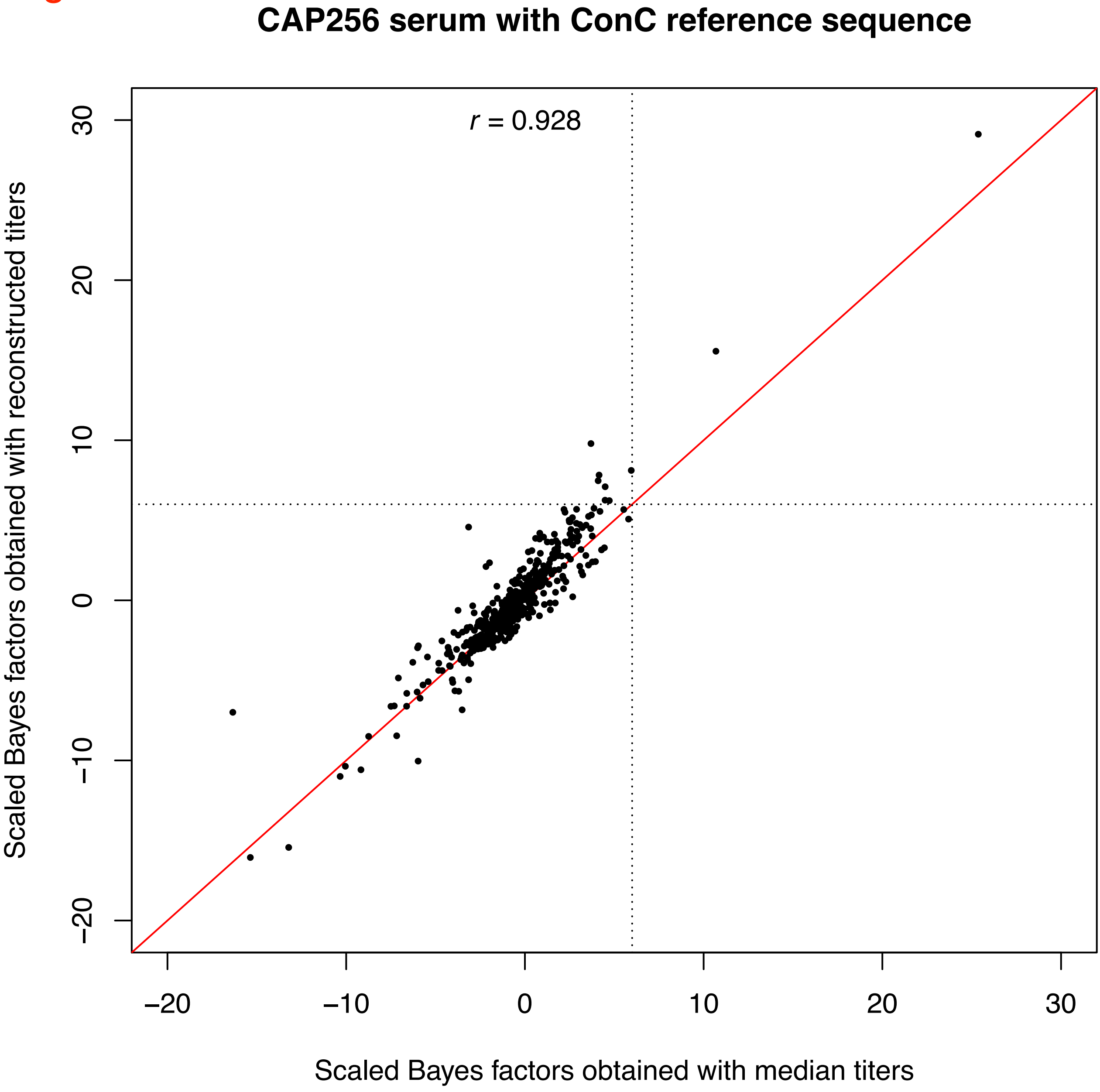

CAP256 serum with ConC reference sequence
r = 0.928
30
20
10
0
−10
−20
Scaled Bayes factors obtained with reconstructed titers
−20
−10
0
10
20
30
Scaled Bayes factors obtained with median titers
