# Supplementary material for: Identification of broadly neutralizing antibody epitopes in the HIV-1 envelope glycoprotein using evolutionary models
Source: Virol J. 2013 Dec 2;10:347. doi: 10.1186/1743-422X-10-347 (PMC4220805; doi:10.1186/1743-422X-10-347)
Supplement: Additional file 2: Table S1 — Reference sequences. Table S2. Estimates used to compute LFDRs. Table S3. Significant associations obtained with the method of Gnanakaran et al. [15]. Table S4. Sites with scaled Bayes factors ≥ 6 using reconstructed titers. [file 1743-422X-10-347-S2.doc]

**Table S1. Reference sequences.**

| Serum | Reference Sequence | Genbank Accession Number |
| --- | --- | --- |
| CAP256 | ConC | DQ401075 |
|  | CAP256 | KF241776 |
|  | CAP210 | DQ435683 |
|  | CAP45 | DQ435682 |
| CAP8 | ConC | DQ401075 |
|  | Q23 | AF004885 |
|  | TRO | AY835445 |
| CAP257 | ConC | DQ401075 |
|  | Q842 | AF407160 |
| CAP255 | ConC | DQ401075 |
|  | CAP255 | EF203982 |
|  | TRO | AY835445 |
|  | Q23 | AF004885 |
| CAP177 | ConC | DQ401075 |
|  | Q23 | AF004885 |
|  | TRO | AY835445 |
| CAP206 | ZM197 | DQ388515 |
|  | CAP206 | EF203967 |
|  | CAP45 | DQ435682 |
|  | Q23 | AF004885 |
|  | COT6 | DQ447266 |
|  | TRO | AY835445 |
| CAP248 | ConC | DQ401075 |
|  | CAP45 | DQ435682 |
|  | DU156 | DQ411852 |

**Table S2**. Estimates used to compute LFDRs.

| Serum | Reference |  |  | $\pi_{0}$ |
| --- | --- | --- | --- | --- |
| CAP8 | ConC | 0.258 | 0.258 | 1.000 |
| CAP177 | ConC | 0.270 | 0.256 | 0.981 |
| CAP206 | ZM197 | 0.302 | 0.273 | 0.960 |
| CAP248 | ConC | 0.257 | 0.261 | 1.000 |
| CAP255 | ConC | 0.252 | 0.244 | 0.989 |
| CAP256 | ConC | 0.276 | 0.238 | 0.950 |
| CAP257 | ConC | 0.287 | 0.276 | 0.985 |

**Table S3. Significant associations obtained with the method of Gnanakaran et al. [15].**

| Serum | HXB2 Position | Sensitive  Classification^†^ | Sensitive  Amino Acid | Resistant Amino Acid |  | *q*-value^‡^ |
| --- | --- | --- | --- | --- | --- | --- |
| CAP256 | 166* | 3 | Arg |  |  | 0.014 |
|  | 169* | 2 | Lys |  |  | 0.015 |
|  | 306 | 2 | Ser |  |  | 0.110 |
|  | 340 | 3 | Glu |  |  | 0.270 |
|  | 360 | 3 | Glu |  |  | 0.006 |
|  | 747 | 2 | Arg |  |  | 0.032 |
| CAP8 | 170 | 3 | Gln |  |  | 0.150 |
|  | 316* | 2 | Thr |  |  | 0.290 |
|  | 775 | 2 |  | Leu |  | 0.260 |
| CAP257 | 440 | 2 |  | Ser |  | 0.300 |
|  | 721 | 1 |  | Phe |  | 0.150 |
| CAP255 | None |  |  |  |  |  |
| CAP177 | 292 | 3 |  | Val |  | 0.120 |
|  | 292 | 3 | Ile |  |  | 0.120 |
|  | 332* | 1 | Asn |  |  | 0.210 |
|  | 332* | 1 |  | Ile |  | 0.210 |
|  | 334* | 1 | Ser |  |  | 0.190 |
| CAP206 | 87 | 3 |  | Glu |  | 0.310 |
|  | 276 | 1 | Asn |  |  | 0.280 |
|  | 336 | 3 | Ser |  |  | 0.280 |
| CAP248 | 171 | 3 |  | Thr |  | 0.280 |

^†^  Definition of a neutralization sensitive virus: (1) ID_50_ ≥ first quartile; (2) ID_50_ ≥ median; (3) ID_50_ ≥ third quartile.

^‡^ Sites with at least one *q* ≤ 1/3 across all amino acids tested for neutralization sensitivity and resistance are shown. Only the smallest *q*-value for each site is reported here.

* Sites with scaled Bayes factors ≥ 6 based on our evolutionary model. As expected, the sensitive amino acids reported here correspond with the reference residues in Table 1.

**Table S4. Sites with scaled Bayes factors ≥ 6 using reconstructed titers.**

| Serum | HXB2 Position | Reference Residue^‡^ | Scaled Bayes Factor | Serum | HXB2 Position | Reference Residue^‡^ | Scaled Bayes Factor |
| --- | --- | --- | --- | --- | --- | --- | --- |
| CAP256 | 2 | Arg | 8.1 | CAP255 | 305 | Lys | 7.7 |
|  | 166*^†^ | Arg | 29.1 |  | 323 | Ile | 6.8 |
|  | 169*^†^ | Lys | 15.6 |  | 332* | Asn | 14.3 |
|  | 234 | Asn | 7.1 |  | 334* | Ser | 13.3 |
|  | 271 | Ile | 6.2 |  | 339 | Asn | 7.3 |
|  | 281 | Leu | 6.2 |  | 602 | Leu | 6.2 |
|  | 309 | Ile | 7.8 |  | 837* | Phe | 11.2 |
|  | 413 | Thr | 9.8 | CAP177 | 209* | Thr | 8.9 |
|  | 739 | Glu | 6.3 |  | 323 | Ile | 7.9 |
|  | 747^†^ | Arg | 7.5 |  | 332*^†^ | Asn | 15.5 |
| CAP8 | 5-6 (insert) | Arg | 6.1 |  | 334*^†^ | Ser | 12.6 |
|  | 170^†^ | Gln | 6.3 |  | 683* | Lys | 8.2 |
|  | 295* | Asn | 12.0 | CAP206 | 150* | Met | 6.5 |
|  | 316*^†^ | Thr | 11.7 |  | 448 | Asn | 6.2 |
|  | 683 | Lys | 7.4 |  | 655* | Lys | 7.9 |
| CAP257 | 166* | Arg | 6.9 | CAP248 | 238 | Pro | 6.8 |
|  | 295* | Asn | 10.5 |  | 624 | Asp | 7.5 |
|  | 309 | Ile | 7.5 |  | 651* | Asn | 9.2 |
|  | 580 | Val | 6.6 |  | 659* | Asp | 9.8 |
|  | 648* | Glu | 6.3 |  | 839 | Ala | 6.6 |
|  | 702* | Leu | 6.7 |  |  |  |  |

* Sites identified using the model with median titers at the ancestral nodes (see Table 1). ^†^ Sites with *q* ≤ 1/3 based on the method of Gnanakaran et al. [15]. ^‡^ Amino acid found to be significantly enriched among sensitive (high titer) viruses based on our evolutionary model.
